# Numb prevents a complete epithelial–mesenchymal transition by modulating Notch signalling

**DOI:** 10.1098/rsif.2017.0512

**Published:** 2017-11-29

**Authors:** Federico Bocci, Mohit K. Jolly, Satyendra C. Tripathi, Mitzi Aguilar, Samir M. Hanash, Herbert Levine, José N. Onuchic

**Affiliations:** 1 Center for Theoretical Biological Physics, Rice University, Houston, TX, USA; 2 Department of Chemistry, Rice University, Houston, TX, USA; 3 Department of Bioengineering, Rice University, Houston, TX, USA; 4 Department of Physics and Astronomy, Rice University, Houston, TX, USA; 5 Department of Biosciences, Rice University, Houston, TX, USA; 6 Department of Clinical Cancer Prevention, UT MD Anderson Cancer Center, Houston, TX, USA

**Keywords:** Numb, hybrid epithelial/mesenchymal phenotype, Notch signalling, circulating tumour cells clusters, epithelial–mesenchymal transition

## Abstract

Epithelial–mesenchymal transition (EMT) plays key roles during embryonic development, wound healing and cancer metastasis. Cells in a partial EMT or hybrid epithelial/mesenchymal (E/M) phenotype exhibit collective cell migration, forming clusters of circulating tumour cells—the primary drivers of metastasis. Activation of cell–cell signalling pathways such as Notch fosters a partial or complete EMT, yet the mechanisms enabling cluster formation remain poorly understood. Using an integrated computational–experimental approach, we examine the role of Numb—an inhibitor of Notch intercellular signalling—in mediating EMT and clusters formation. We show via an mathematical model that Numb inhibits a full EMT by stabilizing a hybrid E/M phenotype. Consistent with this observation, knockdown of Numb in stable hybrid E/M cells H1975 results in a full EMT, thereby showing that Numb acts as a brake for a full EMT and thus behaves as a ‘phenotypic stability factor' by modulating Notch-driven EMT. By generalizing the mathematical model to a multi-cell level, Numb is predicted to alter the balance of hybrid E/M versus mesenchymal cells in clusters, potentially resulting in a higher tumour-initiation ability. Finally, Numb correlates with a worse survival in multiple independent lung and ovarian cancer datasets, hence confirming its relationship with increased cancer aggressiveness.

## 1. Introduction

Epithelial–mesenchymal transition (EMT) and its reverse mesenchymal–epithelial transition (MET) play crucial roles during embryonic development, wound healing and tumour progression [1]. Typically, cells that undergo EMT lose cell–cell adhesion and gain migration and invasion. These bidirectional transitions are rarely ‘all-or-none'. Instead, cells can display one or more hybrid phenotype(s) that possess a mix of epithelial and mesenchymal traits, thereby biasing them to undergo collective cell migration, instead of individual migration enabled by a complete EMT [1]. Collective migration, where cells maintain physical contact with their neighbours, has been considered to be a hallmark of multiple developmental processes such as neural crest migration, branching morphogenesis and wound healing [1]. Recent studies have emphasized that collective cell migration can be a predominant path for cancer metastasis [2]. Collective cell migration can enable the formation of clusters of circulating tumour cells (CTCs) [3]. When compared with individually disseminating CTCs, these clusters are highly resistant to cell death in circulation, possess high tumour-initiation ability, and correlate with a worse clinical outcome across different cancer types [4]. Therefore, deciphering the intracellular and intercellular mechanisms that enable CTC clusters is essential to curb metastatic load.

The formation of clusters of CTC typically requires two conditions. First, individual cells can display a phenotype capable of both adhesion and migration, as is usually found in a hybrid epithelial/mesenchymal (E/M) phenotype [5–8]. Second, such cells must be spatially co-located. It is possible that cells first become hybrid E/M in a random spatial pattern and then dynamically find each other, but this mechanism is much more complex and hence less likely. Thus, we focus our attention to chemical and/or mechanical cell–cell communication mechanisms that can foster the direct formation of clusters via spatial organization; such mechanisms remain relatively less studied.

Previously, we reported that Notch–Jagged signalling may increase the frequency of cells in a hybrid E/M phenotype and their spatial proximity to form clusters of CTCs [9]. Notch signalling is an evolutionarily conserved cell–cell communication signalling pathway comprising a transmembrane receptor, Notch, and two transmembrane ligands, Delta and Jagged. When Notch binds to Delta or Jagged of a neighbouring cell, Notch is cleaved to release Notch intra-cellular domain (NICD) that enters the nucleus, activates the Notch pathway and regulates its target genes [10]. NICD activates the transcription of Notch and Jagged, but represses that of Delta [11]. Thus, Notch–Jagged signalling between two neighbouring cells leads to convergent cell fates (lateral induction) [12,13], whereas Notch–Delta signalling to divergent cell fates (lateral inhibition) [11]. Consequently, neighbouring hybrid E/M can reinforce the stability of hybrid E/M phenotype and lead to the formation of clusters of hybrid E/M cells via Notch–Jagged signalling [9].

Based on this proposed role of Notch–Jagged signalling in inducing and maintaining a hybrid E/M phenotype, we hypothesized that the proteins affecting Notch signalling may modulate the stability of a hybrid E/M phenotype. Here, we focused on Numb and its homologue Numb-like (*Numbl*) that can inhibit Notch signalling through multiple mechanisms [10,14,15]. Also, activated Notch signalling can inhibit Numb and Numb-like, generating a mutually inhibitory feedback loop between Numb/Numb-like and Notch [10]. Identified as a cell-fate determinant in *Drosophila* development, Numb has been since implicated in multiple aspects of cellular homeostasis and tumour progression such as proliferation, apoptosis and stem cell maintenance. Numb-like is much less studied comparatively, and may have partially distinct functions when compared with Numb [16]. However, their effect on Notch has been largely reported to be similar [10].

Here, through a mathematical model for Notch-EMT-Numb signalling axis, we find that Numb or Numbl can prevent the cells from undergoing a complete EMT. This prediction was validated by experiments showing that the knockdown of Numb or Numbl in H1975 lung cancer cells that can maintain a stable hybrid E/M phenotype pushes them towards a complete EMT. Thus, Numb or Numbl may behave as a ‘phenotypic stability factor' (PSF) for a hybrid E/M phenotype. Numb/Numbl can also increase the percentage of hybrid E/M cells in clusters that undergo EMT, potentially enabling the formation of CTC clusters. Consistently, higher levels of Numb or Numbl correlate with poor prognosis, highlighting the aggressive behaviour of a hybrid E/M phenotype.

## 2. Material and methods

### 2.1. Mathematical model of the Notch-epithelial–mesenchymal transition—Numb axis

**Figure 1.**
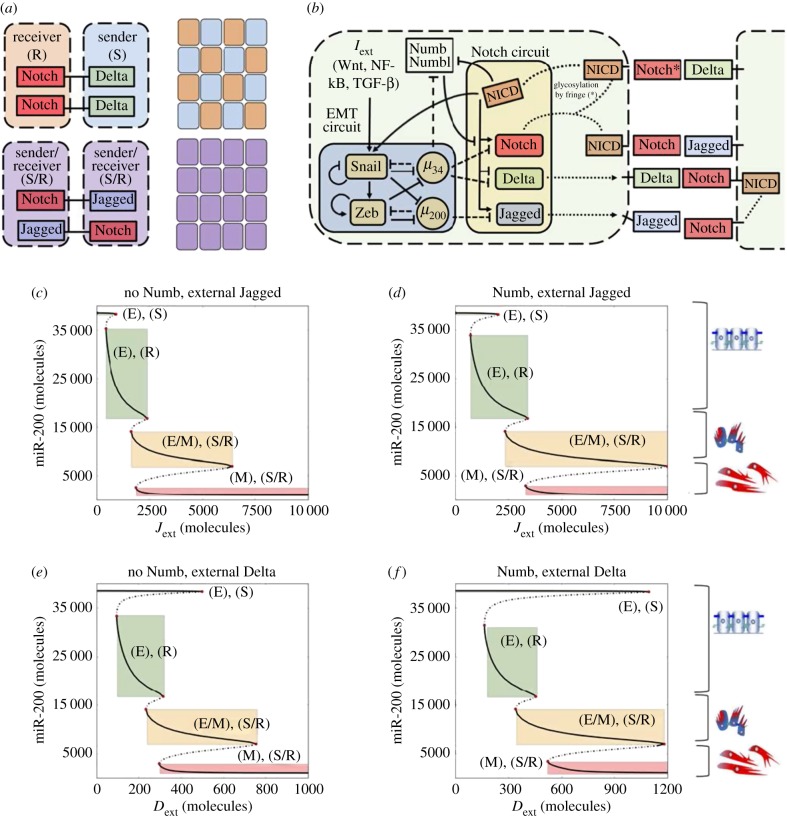
Coupling of Notch signalling with the EMT regulatory circuit and bifurcation curves of miR-200 for Notch-EMT and Numb-Notch-EMT circuits. (*a*) Cells communicating via Notch–Delta signalling exhibit divergent cell fate, one cell being Sender (S, low Notch-high Delta) and the other being receiver (R, high Notch-low Delta). Conversely, cells that interact through Notch–Jagged signalling assume a similar sender/receiver (S/, high Notch–high Jagged) phenotype. At a multi-cell level, Notch–Delta signalling can generate a ‘salt-and-pepper' pattern of sender and receiver cells, while Notch–Jagged signalling generates a uniform distribution of similar S/R cells. (*b*) Schematics of the connection between the EMT regulatory unit and the Notch signalling circuit. The microRNA miR-34 inhibits Notch and Delta, while miR-200 inhibits Jagged and NICD activates Snail. Numb inhibits Notch while being inhibited by NICD. Additionally, miR-34 inhibits Numb. (*c*) Bifurcation curve of the level of miR-200 as a function of external Jagged concentration *J*_ext_ without Numb inhibition acting on Notch. Here, the external concentration of Delta is fixed to zero. Thick and dashed black lines represent stable and unstable steady states, respectively. Cartoons alongside the figure depict which steady states correspond to which EMT phenotypes. Coloured rectangles highlight the interval of stability (parameter on the *x*-axis) and the corresponding level of the microRNA miR-200 (*y*-axis) for the different states. (*d*) Same as (*c*) in the presence of the Numb-related interactions in the system. (*e*) Bifurcation curve of miR-200 as a function of external Delta concentration *D*_ext_ without Numb. The external concentration of Jagged is fixed to zero. (*f*) Same as (*e*) as Numb is inserted in the system. In all simulations, the concentration of external Notch is fixed to Next = 10 000 molecules. Bifurcation curves of all proteins and micro-RNAs in the model are shown in electronic supplementary material, figures S1–S4.

The mathematical model of the Notch–EMT–Numb axis describes the dynamics of the molecular species of the EMT regulatory circuit (miR-34, miR-200, Snail, Zeb), the Notch signalling pathway (Notch receptor, Delta, Jagged, NICD) and Numb according to the schematic of figure 1*a*. The temporal dynamics of the species in the circuit is modelled via a system of ordinary differential equations. The complete set of equations is presented in electronic supplementary material, §S1. Additionally, the post-translational inhibition of Numb by miR-34 is modelled in electronic supplementary material, §S2. Every chemical species is characterized by its own basal production and degradation rate. Furthermore, the production rate of any species can be modulated by transcriptional/translational regulation. Details on how such interactions are modelled can be found in electronic supplementary material, §S1, while all used parameters are given in electronic supplementary material, §S3. Finally, details on the methods used to perform all simulations are discussed in electronic supplementary material, §S4. Details of the experimental protocols used are discussed in §5.

### 2.2. Analysis of clinical data

For all the examined datasets, the pool of patients was divided into two groups according to their expression of Numb being below or above median, and the overall survival and relapse-free survival of the two groups were plotted separately and compared. All survival analysis plots were generated using ProgGeneV2 [17], (http://watson.compbio.iupui.edu/chirayu/proggene/database/?url=proggene).

### 2.3. Numerical calculation and plotting

The single-cell and the multi-cell systems are implemented and solved numerically using the python numerical library PyDsTool [18]. All plots are realized with the plotting library Matplotlib [19]. All source code is freely available on GitHub (https://github.com/federicobocci91/Numb_project).

## 3. Results

### 3.1. Numb inhibits a complete epithelial–mesenchymal transition at a single-cell level

As a first step to investigate the effect of Numb on the dynamics of epithelial-hybrid-mesenchymal transitions, we extend our previously defined mathematical model [9] to include the regulation of Notch by Numb. Both Numb and Numbl form a mutually inhibitory feedback loop with Notch [10] (figure 1*a*), thus, for modelling purposes in the context of this study, we consider Numb and Numbl to be equivalent and group them into one variable—Numb.

As mentioned earlier, Notch signalling takes place when Notch (transmembrane receptor) of one cell binds to Delta or Jagged (transmembrane ligands) of the neighbouring cell(s). Signalling through different ligands, Delta or Jagged, leads to a different phenotypic patterning at a multi-cellular level. Notch–Delta signalling between two cells creates divergent cell fates—one cell behaves as a receiver (high receptor, i.e. Notch, low ligand, i.e. Delta) and the other behaves as a sender (low receptor, i.e. Notch, high ligand, i.e. Delta). Conversely, Notch–Jagged signalling leads to convergent cell fates—both cells behave as hybrid sender/receiver (high receptor, i.e. Notch, high ligand, i.e. Jagged) [11,12] (figure 1*b*). This trait of the Notch–Jagged signalling can contribute to the formation of clusters of hybrid E/M cells by ‘lateral induction' of a hybrid E/M phenotype [9], due to the coupling between Notch and EMT circuits (figure 1*a*), where Notch activates Snail, an EMT-inducing transcription factor, and miR-34 and miR-200 families—guardians of an epithelial phenotype [1]—inhibit Notch, Delta and Jagged [9].

First, we compared the intracellular dynamics of coupled Notch–EMT and Notch–EMT–Numb circuits as a function of fixed levels of external ligands, *J*_ext_ and *D*_ext_, that represent the average concentration of Delta and Jagged available at the surface of the neighbouring cells. Previous work has shown that activation of Notch signalling by either Delta or Jagged can induce a partial or complete EMT in epithelial cells [9,20,21]. Consistently, we observed cells attaining a partial or complete EMT in both cases, i.e. with and without Numb (figure 1*c*–*f*).

In the absence of Numb, at a low external concentration of either ligand, a cell maintains its epithelial phenotype and can behave as either a sender or a receiver—(E), (S) or (E), (R). At higher ligand concentrations, the cell transits to a hybrid E/M state and can act both as sender and receiver—(E/M), (S/R). Eventually, at an even higher concentration of ligands, the cell undergoes a complete EMT—(M), (S/R) (figure 1*c*,*e*). A similar trend is observed in the presence of Numb, but the range of existence of these different states is altered. Numb enlarges the range of *J*_ext_ and *D*_ext_ values for which the (E), (R) and (E), (S) state exist (compare the width of the green rectangle in figure 1*d* versus that in figure 1*c*, and in figure 1*f* versus that in figure 1*e*). Furthermore, the range of values of external ligand concentrations for which the cell maintains a stable hybrid E/M state—(E/M), (S/R)—is increased (compare the width of orange rectangle in figure 1*d* versus that in figure 1*c*, and in figure 1*f* versus that in figure 1*e*). Consequently, cells can maintain a (E/M), (S/R) state at much higher levels of external ligands. Thus, a transition towards a complete EMT state is inhibited. In other words, cells need a stronger stimulus to attain a mesenchymal state (compare the value of *J*_ext_ at the left end of red rectangle in figure 1*d* versus that in figure 1*c*, and the value of *D*_ext_ at the left end of red rectangle in figure 1*f* versus that in figure 1*e*). Altogether, these results indicate that Numb can restrict the progression of a complete EMT, and may stabilize both epithelial and hybrid E/M phenotypes at a single-cell level.

To probe the robustness of these results, we conducted a sensitivity analysis by assessing the change in the interval of stability of the hybrid E/M phenotype resulting from a small variation of the model's parameters. Our results are robust upon parameter variation, albeit a higher sensitivity was observed for some parameters of the original EMT circuit (electronic supplementary material, figures S5 and S6).

Overall, our results suggest that Numb or Numbl can act as a PSF that can stabilize a hybrid E/M phenotype at a single-cell level.

### 3.2. Numb knockdown drives hybrid epithelial/mesenchymal cells to a completely mesenchymal phenotype

To test the prediction of the single-cell model on the action of Numb as PSF for the hybrid E/M phenotype, we knocked down either *Numb* or Numb-like (*Numbl*) in non-small cell lung cancer (NSCLC) H1975 cells that display a stable hybrid E/M phenotype over many passages *in vitro*.

**Figure 2.**
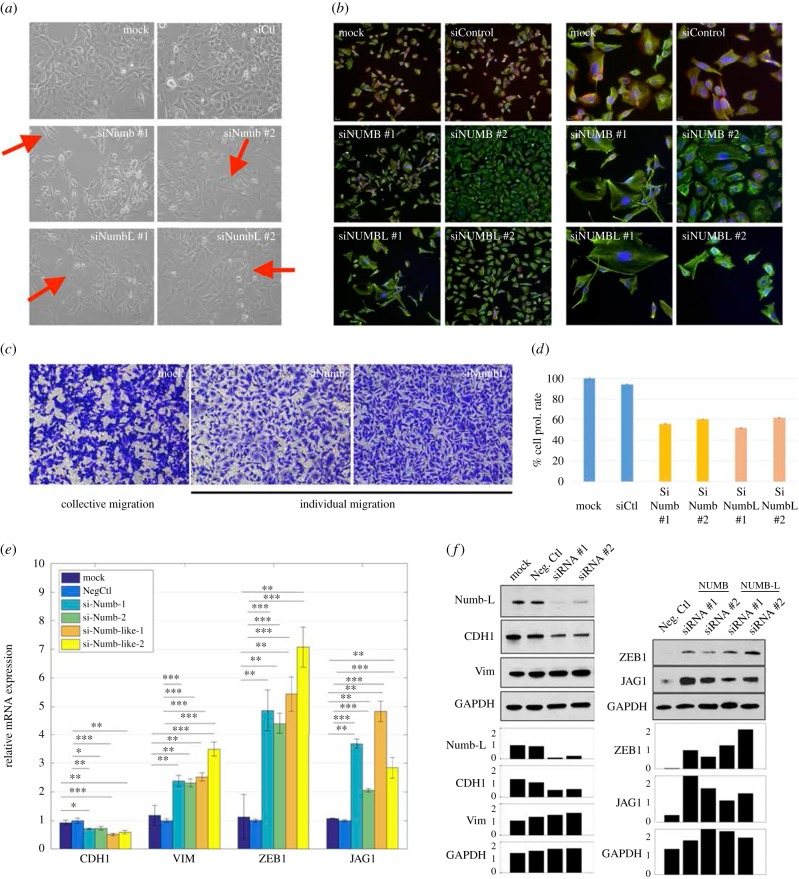
Knockdown of Numb or Numbl induces a full EMT in H1975 cells. (*a*) Bright-field microscopy for mock H1975 cells, H1975 with control siRNA, and H1975 with siRNA against Numb or Numbl. Red arrows indicate visually striking instances of the spindle-like shape that characterize mesenchymal cells, when compared with the more compact shape of the cells in the control (mock, siCtl). (*b*) Immunoflourescence images where red stains for CDH1 (E-cadherin), green for VIM (Vimentin) and blue for DAPI (nucleus). Left panel, magnification 100×, right panel, magnification 200× (*c*) Transwell migration images for mock H1975 cells, and those treated with siRNA against Numb or Numbl. (*d*) Effect of Numb- or Numbl-KD on proliferation of H1975 cells. *N* = 5 for each technical replicate. Error bars represent standard error of mean (s.e.m.). (*e*) RT-PCR measurements of levels of CDH1 (E-cadherin), VIM (vimentin), ZEB1 and JAG1 in cells treated with siRNA either against Numb or Numbl. (*f*) Western blot measurements for CDH1, VIM, ZEB1 and JAG1 in cells treated with either Numb or Numbl. Left panel represents siRNA against NUMBL. Bar charts show measurement quantification done with the software imageJ. Intensities are normalized over the negative control of Numb-L (left) and the siRNA#1 of ZEB (right). Corresponding NUMB results are in electronic supplementary material, figure S7. ‘Neg Ctl' indicates negative control. *p < 0.05, **p < 0.005, ***p < 0.001 using two-tailed paired *t*-test.

Knockdown of Numb or Numbl changed the morphology of H1975 cells to being more spindle-shaped (see cartoon in figure 1*c*,*f* and red arrows in figure 2*a*), and individual cells stained positive only for mesenchymal marker vimentin (VIM) but not for epithelial marker E-cadherin (CDH1), when compared with the control H1975 cells that co-express E-cadherin and vimentin stably over many passages [5] (figure 2*a*,*b*). Moreover, in transwell migration assays, control H1975 cells exhibited collective cell migration, but *Numb*- or *Numbl*-knockdown H1975 cells displayed individual cell migration (figure 2*c*). These observations mimic earlier observations made in multiple contexts such as mammary gland development [14], MCF10A cells [22], MDCK cells [23] and oesophageal cancer cells [24]. Further, knockdown of *Numb* or *Numbl* leads to inhibition of cell proliferation, a trait also typically associated with EMT progression [25] (figure 2*d*). A similar effect on inhibited proliferation was also observed for knockdown of GRHL2—another proposed PSF—in lung [5] and ovarian [26] cancer cells.

Consistently, *Numb*- or *Numbl-*knockdown increased the mRNA and protein levels of (i) mesenchymal marker Vimentin, (ii) EMT-inducing transcription factor ZEB1, and (iii) Notch ligand JAG1. Conversely, mRNA and protein levels of E-cadherin were decreased (figure 2*e*,*f*; electronic supplementary material, S7). Put together, these observations indicate that knockdown of *Numb* or *Numbl* in stable hybrid E/M cells drives them towards a more mesenchymal phenotype, thereby validating our prediction that Numb or Numbl can stabilize a hybrid E/M phenotype and act as a brake on complete EMT progression.

### 3.3. Numb alters the composition of clusters of non-epithelial cells at a tissue level

After evaluating the effect of Numb on EMT at a single-cell level, we compared the dynamics of Notch-EMT and Notch–EMT–Numb circuits at a tissue level by simulating a two-dimensional lattice of 50 × 50 cancer cells communicating with one another via Notch signalling. Specifically, we studied the relative abundance of epithelial (E), hybrid (E/M) and mesenchymal (M) cells and the spatial patterns that these subpopulations form in this lattice, at different production rates of Jagged (*g*_J_) and Delta (*g*_D_), starting from random initial conditions.

**Figure 3.**
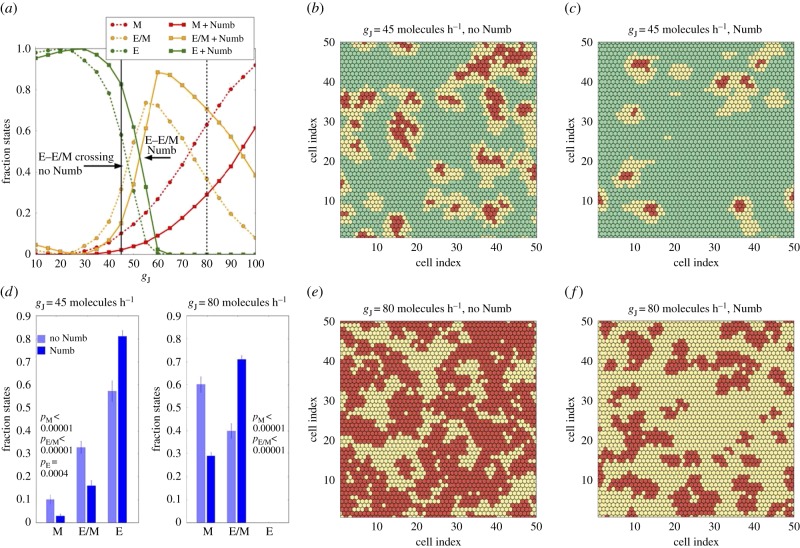
Effect of Numb on tissue patterning for Jagged-dominated Notch signalling. (*a*) Fraction of E, E/M and M cells as a function of the production rate of Jagged (*g*_J_) in the two-dimensional layer of cells in the absence or presence of Numb interactions (dashed and continuous lines, respectively). The vertical continuous and dashed black lines depict the values of *g*_J_ used in (*b*,*c*) and (*e*,*f*), respectively. Numb shifts towards a larger production rate both the crossings between E and E/M cells and between E/M and M cells. (*b*) Snapshot of a two-dimensional layer of cells interacting without Numb for *g*_J_ = 45 molecules h^−1^ corresponding to the E-E/M crossing without Numb. E, E/M and M cells are marked as green, yellow and red, respectively. The colour code is similar for (*c*,*e*,*f*). (*c*) Same as (*b*) for the Notch–EMT–Numb circuit. (*d*) Average fraction of E, E/M and M cells for *g*_J_ = 45 molecules h^−1^ and *g*_J_ = 80 molecules h^−1^. For *g*_J_ = 45 molecules h^−1^, Numb decreases the fraction of both hybrid and mesenchymal cells. At *g*_J_ = 80 molecules h^−1^ all cells have undergone partial or complete EMT, but Numb reduces the fraction of mesenchymal cells. Averages are computed over 10 simulations starting from different randomly chosen phenotype distributions. (*e*) Snapshot for *g*_J_ = 80 molecules h^−1^ in the absence of Numb. (*f*) Same as (*e*) in the presence of Numb. The production rate of Delta is fixed at *g*_D_ = 20 molecules h^−1^ in all plots. The fractions of states and the snapshots were taken after a transient of 120 h starting from the same randomized initial conditions.

We first compared the tissue-level dynamics of Notch-EMT and Notch–EMT–Numb circuits, when cells mainly interact via Notch–Jagged signalling (figure 3). It is worth noting that these results were not collected upon full equilibration of the system, but after a transient time of 5 days, a typical time-scale for EMT. After this time window, we believe that biophysical processes such as altered cell morphology during EMT and consequent cell migration would disrupt the phenotypic patterning that emerges from the model. Notch–Jagged signalling can promote the formation of clusters containing hybrid E/M and M cells [9]. At low levels of Jagged production (*g*_J_ = 45 molecules h^−1^), Notch–Jagged signalling is only weakly activated and thereby weakly induces EMT (see the activation of Snail by NICD in figure 1*a*). In this regime, additional inhibition on this signalling brought by Numb decreases the abundance of both hybrid E/M and mesenchymal cells (figure 3*a*, solid vertical black line), thus halting EMT progression. Consequently, Numb reduces the frequency of clusters containing hybrid E/M and M cells (compare figure 3*c* with figure 3*b*; electronic supplementary material, movies M1 and M2). To quantify the changes induced by Numb, we counted the fraction of epithelial, hybrid and mesenchymal cells over many different simulations (each simulation has slightly different initial conditions). For *g*_J_ = 45 molecules h^−1^, Numb significantly reduces the number of cells in a partial or complete EMT state, and consequently increased those in an epithelial state (figure 3*d*, left).

When comparing Notch–EMT and Notch–EMT–Numb circuits for higher production rates of Jagged (*g*_J_ = 80 molecules h^−1^), a different role of Numb is revealed. In this regime, a strong activation of Notch–Jagged signalling increases the cellular concentration of NICD, thus pushing most cells to either a partial or a complete EMT (figure 3*a*, dashed vertical black line). However, Numb inhibits the accumulation of cells in a complete EMT state by inhibiting Notch signalling and consequently increases those in a hybrid E/M state (compare figure 3*f* with figure 3*e*; electronic supplementary material, movies M3 and M4). This behaviour of Numb as a PSF is reminiscent of its role seen both in H1975 cells (figure 2) and in our single-cell simulations (figure 1). This effect of Numb has been quantified by measuring the change in the fraction of hybrid E/M versus mesenchymal cells in the absence or presence of Numb (figure 3*d*, right) for the case of a large production rate of Jagged that can push approximately 75% cells in a complete EMT state (*g*_J_ = 80 molecules h^−1^).

Finally, to quantify the spatial co-localization of hybrid E/M cells, we counted how many cells adjacent to a hybrid E/M cell exhibited the same, i.e. a hybrid E/M, phenotype (electronic supplementary material, figure S8). For the case of weakly activated Notch–Jagged signalling corresponding to lower *g*_J_ (figure 3*b*,*c*), the average number of hybrid E/M neighbours for a hybrid E/M cell decreased (compare electronic supplementary material, figure S8 middle panel with S8 left panel) due to a decreased total frequency of hybrid E/M cells. However, an increased production of Jagged (figure 3*e*,*f*) can counteract this effect of Numb and consistent with previous reports [9], it can significantly increase the co-localization of hybrid E/M cells (electronic supplementary material, figure S8, right).

**Figure 4.**
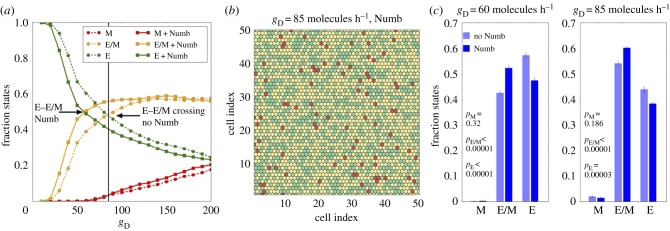
Effect of Numb on tissue patterning for Delta-dominated Notch signalling. (*a*) Fraction of E, E/M and M cells as a function of the production rate of Delta in the two-dimensional layer of cells in the absence or presence of Numb interactions, i.e. Notch–EMT and Notch–EMT–Numb circuits. The vertical continuous line depicts the value of g_D_ used in (*b*) The required production rate to observe a 1 : 1 ratio of E and E/M cells (crossing of green and yellow trajectories) decreases when Notch is inhibited by Numb. (*b*) Snapshot of two-dimensional cell layer for *g*_D_ = 85 molecules h^−1^. E, E/M and M cells are marked as green, yellow and red, respectively. (*c*) Average fraction of E, E/M and M cells for two values of *g*_D_ corresponding to the two crossing points between E and E/M. Averages are computed over 10 simulations starting from different randomly chosen phenotype distributions. The production rate of Jagged is fixed at *g*_J_ = 20 molecules h^−1^ in all plots. The fractions of states and the snapshots were taken after a transient of 120 h starting from the same randomized initial conditions.

Similar to the Notch–Jagged case, we compared the tissue-level spatio-temporal dynamics for Notch–EMT and Notch–EMT–Numb circuits in a lattice of cells that communicate with one another predominantly via Notch–Delta (figure 4). The inhibition of Notch signalling by Numb reduces cellular NICD levels [10], thereby effectively relieving the inhibition of Delta by NICD. This effective increase in the levels of Delta can potentiate Notch signalling in neighbouring cells and thus promote EMT in those cells. As a result, in the case of Notch–EMT–Numb circuit and Delta-dominated signalling, lower basal production levels of Delta (*g*_D_) can enable transitions into the hybrid E/M state, when compared with that required to observe these transitions in the absence of Numb (compare the solid yellow curve with dotted yellow curve in figure 4*a*). Therefore, at a fixed production rate of Delta (*g*_D_), the Notch–EMT–Numb circuit can induce significantly more epithelial cells to attain a hybrid E/M phenotype when compared with that by Notch–EMT circuit (figure 4*c*). Contrary to the case of strong Notch–Jagged signalling, here the increase of the hybrid E/M cell population is mostly due to a decrease in the frequency of epithelial cells. Despite the effect of Numb in altering the ratio of cells in a hybrid E/M and epithelial phenotype, it did not alter the predominant ‘salt-and-pepper' pattern of epithelial and hybrid E/M cells (figure 4*b*; electronic supplementary material, S9). Such pattern formation is a cornerstone of Notch–Delta signalling as observed in multiple biological contexts [11].

Collectively, these results suggest that irrespective of the ligand activating Notch signalling—Delta or Jagged—Numb can increase the number of cells in a hybrid E/M phenotype at both a single-cell and a tissue-level.

**Figure 5.**
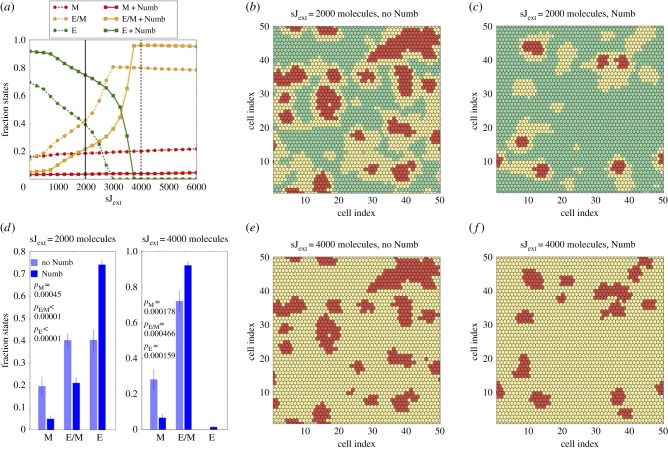
Effect of Numb in the presence of soluble Jagged when Notch signalling is Jagged-dominated. (*a*) Fraction of E, E/M and M cells as a function of the concentration of soluble Jagged sJ_ext_ in the two-dimensional layer of cells in the absence or presence of Numb interactions. Cells in the lattice communicate preferentially through Notch–Jagged signalling (*g*_J_ = 45 molecules h^−1^, *g*_D_ = 20 molecules h^−1^). The vertical continuous and dashed black lines depict the values of sJ_ext_ used in (*b*,*c*) and (*e*,*f*), respectively. (*b*–*c*) Snapshot of the two-dimensional cell layer for sJ_ext_ = 2000 without Numb (*b*) and with Numb (*c*), E, E/M and M cells are marked as green, yellow and red, respectively. The colour code is similar for (*e*) and (*f*). Numb restricts the formation of clusters and decreases the fraction of mesenchymal cells. (*d*) Average fraction of E, E/M and M cells for sJ_ext_ = 2000 and sJ_ext_ = 4000 molecules. In both cases, Numb strongly diminishes both partial and complete EMT. The averages are computed over 10 simulations starting from different randomly chosen phenotype distributions. (*e*–*f*) Snapshot of the two-dimensional cell layer for sJ_ext_ = 4000 without Numb (*e*) and with Numb (*f*): Numb decreases the fraction of mesenchymal cells. Fractions of states and snapshots were measured after a transient of 120 h starting from the configuration of figure 3*b*,*c* as initial conditions for the cases in the absence or presence of Numb, respectively.

After investigating the effect of Numb on the Notch–EMT circuit, we explored the effect of Numb on modulating the paracrine version of Notch signalling, i.e. when cells are exposed to soluble Delta (sD_ext_) or soluble Jagged (sJ_ext_), in addition to membrane-bound ligands (juxtacrine signalling) considered so far in our simulations. Consistent with our results, Numb reduced the frequency of cells in a mesenchymal phenotype in a cohort of cells that were exposed to either soluble ligand (figure 5*a*–*d*; electronic supplementary material, S10–S12). Similar to previous observations (figure 3), an increase in soluble Jagged concentration rescues the cluster frequency, but these clusters predominantly contain hybrid E/M cells and not mesenchymal cells (figure 5*e*,*f*; electronic supplementary material, S12). These effects of Numb on paracrine signalling are more prominent in case of Jagged-dominated juxtacrine signalling instead of Delta-dominated juxtacrine signalling (electronic supplementary material, figures S10 and S12).

In addition, the presence of soluble Jagged in the microenvironment has a crucial consequence on the dynamics of cell fractions in different phenotypes. It can increase the lifetime of transiently observed clusters of hybrid E/M and mesenchymal cells for both Delta-dominated and Jagged-dominated juxtacrine signalling. Without the presence of soluble Jagged, as the Notch–EMT system tends towards a stable equilibrium, hybrid E/M and epithelial cells arrange themselves in a ‘salt-and-pepper' pattern for Delta-dominated signalling. On the other hand, in the case of Jagged-dominated signalling, cells in hybrid E/M and M phenotypes tend to an epithelial switch (elctronic supplementary material, figure S13a,b). The presence of external soluble Jagged stabilizes the hybrid E/M phenotype, thereby further increasing the lifetime of the clusters in the Notch–Jagged signalling case (electronic supplementary material, figure S13c,d).This effect of soluble Jagged in the extracellular environment may help explain how soluble Jagged can drive the cells towards a cancer stem cell phenotype [27] which is often correlated with a hybrid E/M phenotype [1].

It should be noted that soluble Delta- or Jagged-driven signalling is fundamentally different from the formation of intercellular feedback loops between Notch–Delta or Notch–Jagged signalling that are responsible for different patterns formed in Delta-dominated and Jagged-dominated signalling. When soluble ligands—whether Jagged or Delta—activate Notch signalling, the cells only behave as ‘receiver' or ‘target' in case of either ligand, without any tangible feedback on the amount of these soluble ligands. Therefore, Numb similarly affects the dynamics of the system in case of soluble Delta- or soluble Jagged-driven signalling.

### 3.4. External epithelial–mesenchymal transition induction can overcome the inhibition of epithelial–mesenchymal transition by Numb

We next considered the effect of an external EMT inducer such as TGF-β (*I*_ext_) that activates Snail. As shown in the case of Jagged-dominated Notch signalling, high levels of *I*_ext_ significantly increase the number of cells in a fully mesenchymal phenotype (electronic supplementary material, figure S14a). Consistent with our single-cell results, Numb acts as a molecular brake on EMT (compare the dotted curves against solid curves in electronic supplementary material, figure S14a), and therefore a stronger induction of EMT is needed to increase the number of mesenchymal cells. Intriguingly, the frequency of cells in a hybrid E/M phenotype in this case is minimal (electronic supplementary material, figure S14b–d). These results may help explain why ectopic overexpression of ligand-of-Numb X (LNX)—an ubiquitin ligase that targets Numb for degradation—can enhance TGF-β induced EMT [28].

Conversely, when cells communicate predominantly via Notch–Delta signalling, Numb can mildly assist EMT induction and increase the fraction of mesenchymal cells in the population (electronic supplementary material, figure S15a–d). This differential effect of Numb in regulating Notch–Jagged and Notch–Delta signalling is further confirmed by assessing the temporal changes in fraction of epithelial, hybrid E/M and mesenchymal cells (electronic supplementary material, figure S13e,f).

Finally, reproducing the experimental set-up of figure 2, we set up a simulation where all cells in the layer are initially hybrid E/M, and compare the dynamics of the Notch–EMT versus Numb–Notch–EMT circuit in the presence of EMT-induction. Confirming the experimental observation, cells that lack Numb (Notch–EMT case) become mesenchymal on a time-scale of 4–5 days (electronic supplementary material, figure S16).

Finally, we considered the effect of another recently reported feedback regulation in coupled EMT–Notch circuit—the relatively weak inhibition of Numb by miR-34 [29]. Owing to its weak strength, miR-34 only subtly alters the effect of Numb on EMT and Notch signalling (electronic supplementary material, figures S17–S21).

### 3.5. Higher *Numb* or *Numbl* levels predict poor patient survival

The ability of Numb to stabilize a hybrid E/M phenotype and increase the number of hybrid E/M cells in CTC clusters strongly suggested its potential role as a PSF. Given the association of other PSFs such as GRHL2 and *Δ*Np63*α* with poor patient survival [30,31], we next investigated the association of Numb or Numbl with patient survival.

**Figure 6.**
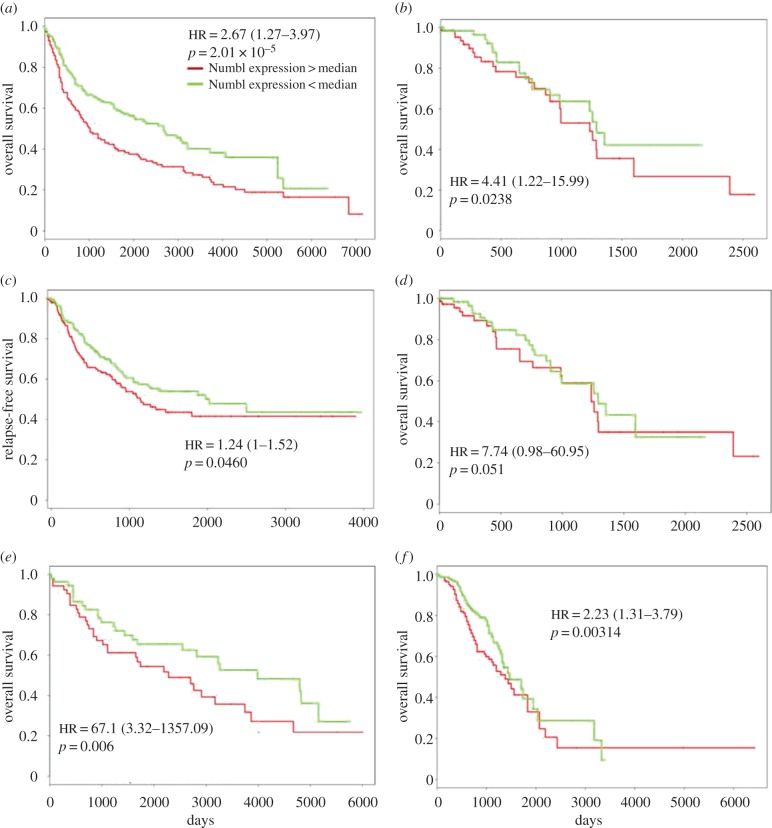
Numb or Numbl can predict poor survival. Survival plots for (*a*) GSE30219 (*n* = 282), (*b*) TCGA-LUAD (*n* = 150), (*c*) GSE 41271 (*n* = 275), (*d*) TCGA-LUAD (*n* = 150), (*e*) GSE 73614 (*n* = 106) and (*f*) GSE9891 (*n* = 276). GSE refers to specific gene expression databases that can be obtained from Gene Expression Omnibus (GEO). HR stands for Hazard Ratio, which, here, refers to the ratio of likelihood of survival of patients with low Numb or Numbl when compared with those with high Numb or Numbl; HR of 1.24 in (*c*) indicates that at a given time-point, patients with high Numbl levels are 24% more likely to express symptoms of relapse when compared with those with low Numbl levels, in that dataset.

High levels of *Numb* or *Numbl* were found to associate with poor overall survival (length of time after the start of treatment of a cancer that the patients are still alive, OS) and relapse-free survival (length of time after the primary treatment of a cancer ends that the patients do not show any symptoms of that cancer, RFS) in multiple independent lung cancer datasets (figure 6*a*–*d*) as well as in ovarian cancer datasets (figure 6*e*,*f*): patients with higher relative levels of *Numb* or *Numbl* were observed to have shorter OS or RFS in these datasets. Our results are consistent with the reported association of high levels of Numb with poor overall survival and post-operative survival across multiple cancer types [32–34].

Low levels of Numb and/or Numbl, indicative of cells that have completely progressed to a mesenchymal phenotype, associate with a better survival, thereby reinforcing the emerging notion that a partial EMT, instead of a full EMT, may be a better marker for tumour aggressiveness [1]. These notions are supported by recent clinical evidence indicating that single-cell migration (a canonical readout of full EMT) happens extremely rarely, if any, in cancer dissemination [2].

## 4. Discussion

Notch signalling pathway is implicated in multiple hallmarks of cancer including metastasis and angiogenesis, and other clinically insuperable aspects such as drug resistance [35]. Here, we investigate how Numb or Numbl—inhibitors of Notch signalling—modulate EMT, a process that can contribute to both metastasis and drug resistance. Our results suggest that either of them can prevent cells from undergoing a complete EMT, and knockdown of Numb or Numbl can induce a full EMT in H1975 cells exhibiting a stable hybrid E/M phenotype. These observations resonate well with recent reports that (i) knockdown of Numb can induce EMT in MCF10A cells [22], (ii) knockdown of Numb or Numbl can induce EMT during mammary gland development [14], and (iii) Numb overexpression led to a loss of mesenchymal markers and features, thereby pushing the cells to an epithelial state [14,22]. Collectively, these results suggest that Numb/Numbl can act as a PSF for a hybrid E/M phenotype, a hypothesis that is bolstered by their individual association with poor patient survival, a trait previously noted for other PSFs such as OVOL2 and GRHL2. The role of Numb or Numbl in predicting poor survival across cancer types reinforces strongly the emerging notion that a hybrid E/M phenotype instead of a full EMT may be the hallmark of tumour aggressiveness [1,36,37].

The effect of Numb/Numbl on tissue-level patterning is reminiscent of glycosyltransferase Fringe that can increase the binding affinity of Notch with Delta, but decrease the affinity with Jagged, thus affecting tissue patterning in a layer of cells [12]. Therefore, both Numb/Numbl and Fringe tend to antagonize Notch–Jagged signalling predominantly (electronic supplementary material, figures S22 and S23). This selective inhibition of Notch–Jagged signalling—an axis involved in drug resistance and colonization [9,38,39]—may help rationalize, at least in part, multiple experimental observations, such as (i) Numb and/or Fringe is/are often lost in many cancer types, including aggressive ones such as basal-like breast cancer [40–42], (ii) *Numbl* knockdown increases chemoresistance and tumorigenic properties in cell lines of different origins—HeLa (cervix), T47D (breast) and AX (sarcoma) [43], (iii) lunatic fringe (*Lfng*) suppresses *in vitro* tumorsphere formation in prostate cancer DU145 cells [40] and (iv) *Numbl* knockdown inhibited the ability of lung cancer cells to form liver metastasis *in vivo* [34].

Importantly, Notch pathway need not be the sole pathway through which Numb modulates EMT. Numb can directly interact with E-cadherin and regulate its membrane localization, as well as control its endocytosis to retain apico-basal polarity in epithelial cells [44,45]. Knockdown of Numb alters E-cadherin localization and polarity complexes such as Par3, and as a result, decreases cell–cell adhesion and increase cell migration [44]. Besides, Numb, but not necessarily Numbl, can stabilize p53 [42] that can activate family members of miR-200 and miR-34 that can restrict EMT and even drive MET [1]. All these aspects of Numb and/or Numbl can be integrated with existing theoretical frameworks to better characterize how Numb affects EMT/MET as well as other traits associated with EMT/MET—immune evasion [46], tumour-initiation potential [1,36] and drug resistance [9,47].

Although we consider Numb and Numbl as equivalent here for mathematical modelling purposes, and observe that knockdown of either of them was sufficient to drive a full EMT in H1975 cells, they may have non-overlapping functions and expression patterns in tissues. For instance, Numb is often associated with asymmetric stem cell division both for developmental stem cell lineages [48] and cancer stem cells (CSCs) [29], but Numbl is symmetrically distributed in daughter cells [49]. Therefore, future modelling efforts will benefit from integrating the different signalling aspects of Numb and Numbl with population-level models of stem cell division. Similarly, consistent with our results, inhibition of either Numb or Numbl can induce Notch activity [43]. However, quantitative differences in effect of Numb versus Numbl, and that in individual versus combined inhibition remain elusive.

To conclude, we found that Numb or Numbl can help in maintaining hybrid E/M phenotype and prevent a full transition to a mesenchymal phenotype, and its knockdown can release the brake for full EMT. Our theoretical framework offers a platform to assess the role of many players that can regulate cellular plasticity in both cell-autonomous and non-cell-autonomous manner, and proposes another target that may potentially break the clusters of tumour cells in a hybrid E/M phenotype—the key drivers of cancer metastasis [1,4].

## Supplementary Material

Supplementary information on the mathematical model and supplementary figures

## Data accessibility

The codes developed for the numerical simulation of the mathematical model are freely available on the github page of FB (federicobocci91). To access experimental data, please contact Prof. Samir Hanash (shanash@mdansderon.org).
