# Supplementary material for: Numb prevents a complete epithelial–mesenchymal transition by modulating Notch signalling
Source: J R Soc Interface. 2017 Nov 29;14(136):20170512. doi: 10.1098/rsif.2017.0512 (PMC5721160; doi:10.1098/rsif.2017.0512)
Supplement: Supplementary information on the mathematical model and supplementary figures [file rsif20170512supp1.pdf]

Numb prevents a complete Epithelial-Mesenchymal Transition by modulating Notch signalling

Supplementary Information

Federico Bocci[a,b,*], Mohit Kumar Jolly[a,*], Satyendra C. Tripathi[f*], Mitzi Aguilar[f], Samir M Hanash[f,#], Herbert Levine[a,c,d,e,#] and José N. Onuchic[a,b,d,e,#]

Center for Theoretical Biological Physics[a] and Department of Chemistry[b], Bioengineering[c], Physics and Astronomy[d] and Biochemistry and Cell Biology[e], Rice University, Houston, TX USA;

Department of Clinical Cancer Prevention, UT MD Anderson Cancer Center, Houston, TX, USA

*These authors equally contributed to this work.

#Corresponding authors: Samir M Hanash (shanash@mdansderon.org), Herbert Levine (hl34@rice.edu), Jose N Onuchic (jnonuchic@rice.edu)

1. Notch-Numb-EMT circuit

The scheme of Fig. 1a is implemented by generalizing the mathematical model of Boareto et al(1), that couples the theoretical framework of the Notch-Delta-Jagged cell-cell signalling pathway by Boareto, Kumar et al(2,3) with the model of the EMT core regulatory circuit developed by Lu et al(4). The dynamics of Notch (N), Delta (D), Jagged (J), Numb (U) and NICD (I) are described by:

$$(1)\ \frac{dN}{dt} = k_P g_N H^{S+}(I,N) H^{S-}(U,N) P_l(\mu_{34},2) - N\big[(k_{cD}D + k_{cJ}J\big) + \big[(k_{ct}D_{ext} + (k_{tJ}J_{ext})\big] - \gamma N$$

$$(2)\ \frac{dD}{dt} = k_P g_D H^{S-}(I,D) P_l(\mu_{34},3) - D\ (k_{cD}N + k_{tD}N_{ext})\ - \gamma D$$

$$(3)\ \frac{dJ}{dt} = k_P g_J H^{S+}(I,J) P_l(\mu_{200},5) - J\ (k_{cJ}N + k_{tJ}N_{ext})\ - \gamma J$$

$$(4)\ \frac{dU}{dt} = U_0 H^{S-}(I,U)\ - \gamma_U U$$

$$(5)\ \frac{dI}{dt} = N(k_{ct}D_{ext} + k_{tJ}J_{ext}) - \gamma_I I$$

where g_N, $g_D$and g_J are the transcription rates of Notch, Delta and Jagged and $k_P$is the translation rate. U_0 is the production rate of Numb. γ is the degradation rate of Notch, Delta and Jagged and γ_U is the degradation rate of the newly introduced Numb; NICD degrades at its own rate γ_I. The transcriptional activation/inhibition that a species (R) exerts on another component (X) is modelled via shifted Hill functions:

$$(6)\ H^S(R,X) = \frac{1}{1+\left(\frac{R}{R_{0,X}}\right)^{n_{R,X}}} + \lambda_{R,X}\frac{\left(\frac{R}{R_{0,X}}\right)^{n_{R,X}}}{1+\left(\frac{R}{R_{0,X}}\right)^{n_{R,X}}}$$

where R_0 is the half-maximal concentration and $\lambda_{R,X}$ is the fold change of the production rate ($\lambda_{R,X} < 1$ for inhibition (H^{S-}), $\lambda_{R,X} > 1$ for activation, (H^{S+})). In the model, NICD activates Notch and Jagged ($H^{S+}(I,N)$, $H^{S+}(I,J)$) and inhibits Delta and Numb ($H^{S-}(I,D), H^{S-}(I,U)$), respectively. Also, the inhibitory effect that Numb exerts on Notch is modelled via $H^{S-}(U,N)$. The parameters k_{cD}, k_{cJ} and k_{tD}, k_{tJ} model the cis-inhibition and the trans-activation of the Notch-Delta and Notch-Jagged interactions,

respectively. The binding of Notch with Delta and Jagged can be altered by the glycosyltransferase Fringe, which increases the affinity of Notch to Delta but decreases the affinity to Jagged(5). Since Fringe is activated by NICD, this effect was modelled as an effective activation/inhibition of the cis- and trans-binding constants of Delta/Jagged with Notch via shifted hill functions, namely $k_{c/t,D/J} = k_{c/t}H^S(I, D/J, \lambda_{I,D/J})$. $N_{ext}, D_{ext}, J_{ext}$ represent either the external concentration of Notch and ligands for the single cell system or the overall level of the proteins available for binding in the neighbouring cells in the multi-cell case.

Also, Notch receptor and ligands could be degraded due to post-translational regulation upon binding with micro-RNAs (miR-200, miR-34). The function $P_l(\mu, n)$ represents the post-translational inhibition that a micro-RNA species μ exerts on a transcription factor or receptor/ligand, where n is the number of corresponding available binding sites. $P_y(\mu, n)$ describes the corresponding decrease in the level of micro-RNA μ due to the degradation of the micro-RNA/mRNA complex. These functions are the standard modelling treatment of micro-RNA post-translational interactions in the MBC framework (microRNA-Based-Chimeric circuits) developed by Lu et al(4), and are defined as:

$$(7)\ \ P_l(\mu, n) = \frac{L(\mu, n)}{Y_m(\mu, n) + k_m}$$

$$(8)\ \ P_y(\mu, n) = \frac{Y_\mu(\mu, n)}{Y_m(\mu, n) + k_m}$$

where $L(\mu, n)$ is the total translation rate of the ligand/receptor, $Y_m(\mu, n)$ is the total active degradation rate of the messenger RNA of the ligand/receptor and $Y_\mu(\mu, n)$ is the total degradation rate of the micro-RNA:

$$(9)\ \ L(\mu, n) = \sum_{i=0}^{n} l_i {C_i}^n {M_i}^n(\mu)$$

$$(10)\ \ Y_m(\mu, n) = \sum_{i=0}^{n} \gamma_{mi} {C_i}^n {M_i}^n(\mu)$$

$$(11)\ \ Y_\mu(\mu, n) = \sum_{i=0}^{n} \gamma_{\mu i} {C_i}^n {M_i}^n(\mu)$$

In eqs. (9)-(11), l_i, γ_{mi} and $\gamma_{\mu i}$ are individual rates for the case of i micro-RNAs bound to the protein. Also, ${C_i}^n$ is the number of arrangements of i molecules of micro-RNA in n binding sites:

$$(12)\ \ {C_i}^n = \frac{n!}{i!\,(n-i)!}$$

and

$$(13)\ \ {M_i}^n(\mu) = \frac{\left(\frac{\mu}{\mu_0}\right)^i}{\left(1+\frac{\mu}{\mu_0}\right)^n}$$

where μ_0 is a threshold for micro-RNA concentration. Please see the Supplementary Information of Lu et al(4) for a more thorough discussion of the MBC framework.

The dynamics of the species of the EMT circuit, miR-200 (μ_{200}), miR-34 (μ_{34}), Zeb (Z) and Snail (S), are given by:

$$(14)\ \ \frac{d\mu_{200}}{dt} = g_{\mu_{200}} H^S(Z,\mu_{200}) H^S(S,\mu_{200}) - g_Z H^S(Z,Z) H^S(S,Z) P_y(\mu_{200},6) - g_J H^S(I,J) P_y(\mu_{200},5) - \gamma_{\mu_{200}}\mu_{200}$$

$$(15)\ \ \frac{d\mu_{34}}{dt} = g_{\mu_{34}} H^S(S,\mu_{34}) H^S(Z,\mu_{34}) - g_S H^S(S,S) H^S(I,S) H^S(I_{ext},S) P_y(\mu_{34},2) - g_D H^S(I,D) P_y(\mu_{34},3) - g_N H^S(I,N) P_y(\mu_{34},2) - \gamma_{\mu_{34}}\mu_{34}$$

$$(16)\ \ \frac{dZ}{dt} = k_P g_Z H^S(Z,Z) H^S(S,Z) P_y(\mu_{200},6) - \gamma_Z Z$$

$$(17)\ \ \frac{dS}{dt} = k_P g_S H^S(S,S) H^S(I,S) H^S(I_{ext},S) P_y(\mu_{34},2) - \gamma_S S$$

Here, $g_{\mu_{200}}$, $g_{\mu_{34}}$, $g_Z$and g_S are the production rates of miR-200, miR-34, Zeb and Snail, while $\gamma_{\mu_{200}}$, $\gamma_{\mu_{34}}$, $\gamma_Z$and γ_S are the degradation rates, and the functions H^S, $P_l(\mu,n)$ and $P_y(\mu,n)$ were already discussed when defining the Notch circuit.

Please refer also to the Supplementary Information of Boareto et al(1) for the derivation of the equations.

2. Implementation of the miR-34 inhibition on Numb

The inhibition of miR-34 on Numb is introduced by explicitly considering the dynamics of the Numb protein U and its respective mRNA m_U:

$$(18)\quad \frac{dm_U}{dt} = g_U H^S(I,U) - m_U Y_m(\mu_{34},1) - \gamma_{m_U} m_U$$

$$(19)\quad \frac{dU}{dt} = k_P{}^U m_U L(\mu_{34},1) - \gamma_U U$$

Here, γ_U and γ_{m_U} are the production and degradation rates of mRNA, respectively, and $k_P{}^U$ is the translation rate of Numb. The functions $Y_m(\mu_{34},1)$ and $L(\mu_{34},1)$ represent active mRNA degradation and translation rates for the microRNA μ_{34} binding to mRNA m_U where there is n=1 binding sites for μ_{34} on m_U. Since translation is much faster than all other processes, quasi-steady approximation is assumed for mRNA dynamics $\frac{dm_U}{dt} = 0$, yielding:

$$(20)\quad m_U = \frac{g_U H^S(I,U)}{Y_m(\mu_{34},1) + \gamma_{m_U}}$$

Thus, the equation for U becomes:

$$(21)\quad \frac{dU}{dt} = k_P{}^U g_U H^S(I,U) \frac{L(\mu_{34},1)}{Y_m(\mu_{34},1) + \gamma_{m_U}} - \gamma_U U = k_P{}^U g_U H^S(I,U) P_l(\mu_{34},1) - \gamma_U U$$

where in the last equation, we exploited the definition of $P_l(\mu_{34},1)$. Similarly, the additional active degradation term in the dynamics of μ_{34} is $g_U H^S(I,U) P_y(\mu_{34},1)$, yielding the updated equation:

$$(22)\quad \frac{d\mu_{34}}{dt} = g_{\mu_{34}} H^S(S,\mu_{34}) H^S(Z,\mu_{34}) - g_S H^S(S,S) H^S(I,S) H^S(I_{ext},S) P_y(\mu_{34},2)$$

$$-g_D H^S(I,D) P_y(\mu_{34},3) - g_N H^S(I,N) P_y(\mu_{34},2) - g_U H^S(I,U) P_y(\mu_{34},1) - \gamma_{\mu_{34}} \mu_{34}$$

3. Parameters estimation

The parameters of the Notch circuit are taken from the model of Boareto et al(2) without changes, and are presented in Table 1. Similarly, the parameters related to the EMT circuit are presented in Table 2 and were taken from the original work of Lu et al(4), as well as the degradation/translation rates for post-translational regulation by micro-RNAs presented in table 3. While the interested reader can find detailed information about the estimation of these parameters in the original paper, the general principles used to determine their numerical value were:

- Degradation rates were estimated from literature;
- Production rates were chosen to maintain a level of receptor and ligands of the order of a few thousand units per cell and a level of a few hundreds of thousands of micro-RNAs;
- Hill fold-changes were estimated using Western Blotting with knockdown of the gene/micro-RNA/protein of interest, while Hill coefficients and thresholds could be estimated by looking at expression measurements for different inducer/inhibitor concentrations.
- Trans-activation and cis-inhibition coefficients for receptor-ligand interactions were chosen under the assumption that Notch dynamics is slower than EMT dynamics.

The newly introduced parameters that relates to Numb are presented in Table 4. The rate of degradation of Numb γ_U was taken as equal to the other proteins in the system (Notch, Delta, Jagged). The production rate was estimated by imposing an equilibrium reference level of Numb molecules in the cell of 10^6 in the absence of interactions (i. e. without inhibition from Notch). The inhibition of Numb by Notch is weak(6), thus we chose $\lambda_{I,U} = 0.5$. For this choice of $\lambda_{I,U}$ the steady-state level of Numb lies within the range $[0.5\ 10^6, 10^6]$, thus we chose a switching level $U_N = 0.75\ 10^6$ molecules for the Hill function representing the inhibition of Notch by Numb. The inhibition of Notch by Numb is weak as well(6), thus we chose $\lambda_{I,U} = 0.8$. The Hill coefficients for the Numb-Notch double negative feedback were both assumed to be 2, while the Hill threshold for the inhibition of Numb by NICD equals the threshold of NICD for Notch,

Delta and Jagged.

The rate of translation of Numb ${k_P}^U$ is equal to all other proteins in the system (Notch, Delta, Jagged). The production rate of mRNA g_U was inferred with an internal consistency requirement: imposing the absence of inhibition by miR-34 on Numb (i.e. n=0 binding sites for miR-34 on m_U) yields $L(\mu_{34}, 0) = 1$ and $Y_m(\mu_{34}, 0) = 0$, so:

$$\frac{dm_U}{dt} = g_U H^S(I, U) - \gamma_{m_U} m_U$$

$$\frac{dU}{dt} = {k_P}^U m_U - \gamma_U U$$

so, quasi-steady approximation of mRNA m_U yields:

$$\frac{dU}{dt} = \frac{{k_P}^U g_U}{\gamma_{m_U}} H^S(I, U) - \gamma_U U = U_0 H^S(I, U) - \gamma_U U$$

where the last term is the dynamics of Numb in the absence of inhibition by miR-34. Then $g_U = \frac{U_0 \gamma_{m_U}}{{k_P}^U} = 100\ mRNA\ h^{-1}$, where $\gamma_{m_U} = 0.1\ h^{-1}$ (same as degradation of transcription factors and ligands).

Parameter group	Parameter	Value	Dimensions
Degradation	$\gamma_N, \gamma_D, \gamma_J, \gamma_I, \gamma_U$	$0.1, 0.1, 0.1, 0.5, 0.1$	h^{-1}
Production	g_N, g_D, g_J, U_0	$8, 20(\#), 70(\#), 10^5$	$mRNA\ h^{-1}$
Hill coefficient	$n_{I,N}, n_{I,D}, n_{I,J}, n_{I,U}, n_{U,N}$	$2, 2, 5, 2, 2$	$Dimensionless$
Hill fold-change	$\lambda_{I,N}, \lambda_{I,D}, \lambda_{I,J}, \lambda_{I,U}, \lambda_{U,N}$	$2, 0, 2, 0.6, 0.8$	$Dimensionless$
Hill threshold	$I_{0,N}, I_{0,D}, I_{0,J}, I_{0,U}, U_{0,N}$	$200, 200, 200, 200, 10^5$	$Molecules$
Binding rates	k_T, k_C	$1\ 10^{-4}, 1\ 10^{-5}$	$h^{-1} Molecules^{-1}$
Fringe	$n_F, \lambda_{F,D}, \lambda_{F,J}$	$1.0, 3.0, 0.3$	$Dimensionless$

Table 1. Parameters of the Notch circuit. (#) refers to parameters that were modified in the multi-cell figures (Fig. 3-4-5).

Parameter group	Parameter	Value	Dimensions
Degradation	$\gamma_S, \gamma_Z, \gamma_{\mu_{34}}, \gamma_{\mu_{200}}$	$0.1, 0.1, 0.5, 0.5$	h^{-1}
Production	$g_S, g_Z, g_{\mu_{34}}, g_{\mu_{200}}$	$90, 11, 1350, 2100$	$mRNA\ h^{-1}$
Hill coefficient	$n_{Z,\mu_{34}}, n_{Z,\mu_{200}}, n_{S,\mu_{34}}, n_{S,\mu_{200}}, n_{Z,Z}, n_{S,S}, n_{S,Z}, n_{I,S}, n_{Iext,S}$	$2, 3, 1, 2, 2, 1, 1, 2, 2$	$Dimensionless$
Hill fold-change	$\lambda_{Z,\mu_{34}}, \lambda_{Z,\mu_{200}}, \lambda_{S,\mu_{34}}, \lambda_{S,\mu_{200}}, \lambda_{Z,Z}, \lambda_{S,S}, \lambda_{S,Z}, \lambda_{I,S}, \lambda_{Iext,S}$	$0.2, 0.1, 0.1, 0.1, 7.5, 0.1, 10, 6.5, 6.5$	$Dimensionless$
Hill threshold	$Z_{0,\mu_{34}}, Z_{0,\mu_{200}}, S_{0,\mu_{34}}, S_{0,\mu_{200}}, Z_{0,Z}, S_{0,S}, S_{0,Z}, I_{0,S}, Iext_{0,S}$	$600K, 220K, 300K, 180K, 25K, 200K, 180K, 300, 300$	$Molecules$ $(K = 1000)$

Table 2. Parameters of the EMT circuit.

Parameter group	Parameter	Value	Dimensions
Translation rate	l_i	$1.0, 0.6, 0.3, 0.1, 0.05, 0.05, 0.05$	
mRNA degradation rate	γ_{mi}	$0, 0.04, 0.2, 1.0, 1.0, 1.0, 1.0$	h^{-1}
micro-RNA degradation rate	$\gamma_{\mu i}$	$0, 0.005, 0.05, 0.5, 0.5, 0.5, 0.5$	h^{-1}

Table 3. Parameters for translation, mRNA degradation and micro-RNA degradation upon protein-micro-RNA binding.

Parameter Description	Parameter	Value	Dimensions
Production rate	U_0	10^5	$Molecules\ h^{-1}$
Inhibition on Numb – fold change	$\lambda_{I,U}$	0.5	$Dimensionless$
Inhibition on Numb – Hill coefficient	n_U	2	$Dimensionless$
Inhibition on Numb – Threshold	$I_{0,U}$	200	$Molecules$
Inhibition on Notch – fold change	$\lambda_{U,N}$	0.8	$Dimensionless$
Inhibition on Notch – Hill coefficient	n_N	2	$Dimensionless$
Inhibition on Notch – Threshold	$U_{0,N}$	$0.75\ 10^6$	$Molecules$
Degradation rate	γ_U	0.1	h^{-1}
Transcriptional production rate	${k_P}^U$	100	$Proteins\ per\ mRNA\ h^{-1}$
mRNA production rate	g_U	100	$mRNA\ h^{-1}$

Table 1 Parameters of the Numb-related interactions.

4. Simulation details

In the single cell model, the isolated cell is described by the levels of all proteins, transcription factors (TFs) and micro-RNAs (miRs) presented in Fig. 1a, always expressed in number of molecules. Additionally, the cell can be exposed to an external level of Notch receptor and ligands Delta and Jagged (N_{ext}, D_{ext}, J_{ext}) to mimic an incoming signal from neighboring cells. The cell is classified as epithelial (E), hybrid (E/M) or mesenchymal (M) according to its level of micro-RNA 200 (miR-200 <5000 molecules: M; 5000<miR-200<15000: E/M; miR-200>15000: E). This classification still holds true even when parameters of the model are modified (Fig. S24). The bifurcation diagrams for the single cell model were realized with the PyDSTool Python library(7).

The multi-cell model extends the computational framework of the Notch-Numb-EMT axis to a two-dimensional 50x50 layer of cells. The level of all molecular players (Notch, Delta, Jagged, NICD, Numb, miR-200, miR-34, Zeb, Snail) are described by the same set of equations of the single cell model. The layer of cells is modelled as a two-dimensional hexagonal lattice. Each hexagon represents a cell that is surrounded by six neighbouring cells with whom it communicates through the Notch pathway. At any given time t, the amount of Notch receptors and ligands available to bind to one cell's receptors and ligands are given by:

$$N_{ext} = \sum N_n$$
$$D_{ext} = \sum D_n + D_{sol}$$
$$J_{ext} = \sum J_n + J_{sol}$$

where N_n, D_n, J_n represent the amount of Notch receptor and ligands of the neighbor cells n, and the sum runs along all 6 nearest neighbors. D_{sol} and J_{sol} represent the amount of soluble Delta and Jagged present in the environment. Soluble ligands have been reported in mediating multiple aspects of cancer progression such as Cancer Stem Cells(8) - a subpopulation of cells that shares traits with cells undergoing EMT(9). When present (as in Fig. 5), this term is constant for the duration of the simulation. Also, cells in the tissue can be exposed to an external EMT-inducer that directly activates Snail (see Snail equation). The equations governing the dynamics of the multi-cell Notch-Numb-EMT circuit were integrated numerically using an

explicit Euler method with a time step of $0.1\,h$ and imposing periodic boundary conditions. Initial conditions for the levels of all chemical species in each cell were chosen randomly. The different variables were sampled homogeneously from the whole intervals allowed by the bifurcation diagrams of Fig. S1-S4.

5. Experimental procedures

Cell culture and transfection

H1975 cells were obtained from the American Type Culture Collection. All cells grew in RPMI 1640 with 10% fetal bovine serum and a 1% penicillin/streptomycin cocktail (Thermo Fisher Scientific, Waltham, MA). Cell lines were cultured continuously for 6 months or less. Cell lines were validated at the MD Anderson Sequencing and Microarray Facility using short-tandem repeat DNA fingerprinting and routinely checked for mycoplasma by PCR (PromoKine). Cells were transfected at a final concentration of 50 nM siRNA using Lipofectamine RNAiMAX (Thermo Fisher Scientific) according to the manufacturer's instructions using following siRNAs: siControl (Silencer Select Negative Control No. 1, Thermo Fisher Scientific), siNumb #1 (SASI_Hs01_00245422, Sigma-Aldrich, St.Louis, MO), siNumb #2 (SASI_Hs02_00305013, Sigma-Aldrich, St.Louis, MO),), siNumb-L #1 (SASI_Hs01_00103891, Sigma-Aldrich), siNumb-L #2 (SASI_Hs01_00103892, Sigma-Aldrich). siRNA treatment was done for 48 hrs and then replated on coverslips for 24 hrs and then fixed.

RT-PCR

Total RNA was isolated following manufacturer's instructions using RNAeasy kit (Qiagen). cDNA was prepared using iScript gDNA clear cDNA synthesis kit (Bio-Rad). A TaqMan PCR assay was performed with a 7500 Fast Real-Time PCR System using TaqMan PCR master mix, commercially available primers, and FAM™-labelled probes for CDH1, VIM, ZEB1, NUMB, NUMBL, and JAG1 and VIC™-labelled probes for 18S, according to the manufacturer's instructions (Life Technologies). Each sample was run in triplicate. Ct values for each gene were calculated and normalized to Ct values for 18S (ΔCt). The ΔΔCt values were then calculated by normalization to the ΔCt value for control.

Western Blotting Analysis and Immunofluorescence

Cells were lysed in RIPA lysis assay buffer (Pierce) supplemented with protease and phosphatase inhibitor. The samples were separated on a 4–15% SDS-polyacrylamide gel (Biorad). After transfer to PVDF membrane, probing was carried out with primary antibodies and subsequent secondary antibodies. Primary antibodies were purchased from the following commercial sources: anti-CDH1 (1:1000; Cell Signalling Technology), anti-vimentin (1:1000; Cell Signalling Technology), anti-Zeb1 (1:1000; Cell Signalling Technology), anti-JAG1 (1:1000; Abcam), anti-Numb (1:1000; Abcam), anti-Numb-L (1:1000; Abcam) and anti-GAPDH (1:10,000; Abcam). Membranes were exposed using the ECL method (GE Healthcare) according to the manufacturer's instructions. For immunofluorescence, cells were fixed in 4% paraformaldehyde, permeabilized in 0.2% Triton X-100, and then stained with anti-CDH1 (1:100; Abcam) and anti-vimentin (1:100; Cell Signalling Technology). The primary antibodies were then detected with Alexa conjugated secondary antibodies (Life technologies). Nuclei were visualized by co-staining with DAPI.

Trans-well migration assay

H1975 cells were grown in 6-well plates and treated with siRNAs for Numb and Numb-L for 24 hours. After 48hrs of siRNA knockdown, cell monolayers were harvested, counted and cell concentration was adjusted to 2×10^4 viable cells/200 µl of serum free medium. The cell suspension was seeded on top of a Transwell insert with 0.8 µm pore diameter (Millipore) and placed on a 24 well cell culture plate. At the bottom of the Transwell insert, 10% fetal bovine serum was added as chemo-attractant and plate was incubated at 37°C for 18 hours. Medium was aspirated from assay plate and Transwell inserts. Cells that did not migrate were removed by gently swabbing the inside of each insert using cotton swabs. After 3 washes with PBS, cells were fixed and stained with a 0.5% crystal violet solution for 10 minutes. After the cells have been stained, the inserts were washed thoroughly with water until the water runs clear. Inserts

were dried completely before visualizing with a microscope. The image is from 72 hrs experiment just to match the time course of the Immunofluorescence (IF) experiments.

Proliferation assay

MTS assay (CellTiter 96 Aqueous One Solution Cell Proliferation Assay, Promega) was performed to assess the cell viability after 72 hours and 96 hours. The experiments were repeated 3 times each with five technical replicates. The error bar represents the % standard deviation of the MTS values normalized with controls. We followed the standard protocol as per the manufacturer's instruction (Promega, CellTiter 96® AQueous One Solution Cell Proliferation Assay).

References

1. Boareto M, Jolly MK, Goldman A, Pietilä M, Mani SA, Sengupta S, et al. Notch-Jagged signalling can give rise to clusters of cells exhibiting a hybrid epithelial/mesenchymal phenotype. J R Soc Interface. 2016;13(118):20151106.

2. Boareto M, Jolly MK, Lu M, Onuchic JN, Clementi C, Ben-Jacob E. Jagged--Delta asymmetry in Notch signaling can give rise to a Sender/Receiver hybrid phenotype. Proc Natl Acad Sci. 2015;112(5):E402--E409.

3. Jolly MK, Boareto M, Lu M, Onuchic JN, Clementi C, Ben-Jacob E, et al. Operating principles of Notch--Delta--Jagged module of cell--cell communication. New J Phys. 2015;17(5):55021.

4. Lu M, Kumar M, Levine H, Onuchic JN, Ben-jacob E. MicroRNA-based regulation of epithelial-hybrid mesencymal fate determination. Proc Am Thorac Soc. 2013;110(45):18144–18149.

5. Kopan R, Ilagan MXG. The Canonical Notch Signaling Pathway: Unfolding the Activation Mechanism. Cell. 2009 Apr;137(2):216–33.

6. Zhang Y, Li F, Song Y, Sheng X, Ren F, Xiong K, et al. Numb and Numbl act to determine mammary myoepithelial cell fate, maintain epithelial identity, and support lactogenesis. FASEB J. 2016;30(10):3474–88.

7. Clewley R. Hybrid models and biological model reduction with PyDSTool. PLoS Comput Biol. 2012;8(8):e1002628.

8. Lu J, Ye X, Fan F, Xia L, Bhattacharya R, Bellister S, et al. Endothelial cells promote the colorectal cancer stem cell phenotype through a soluble form of Jagged-1. Cancer Cell. 2013 Feb;23(2):171–85.

9. Mani SA, Guo W, Liao M-J, Eaton EN, Ayyanan A, Zhou AY, et al. The epithelial-mesenchymal transition generates cells with properties of stem cells. Cell. 2008;133(4):704–15.

Supplementary Figures

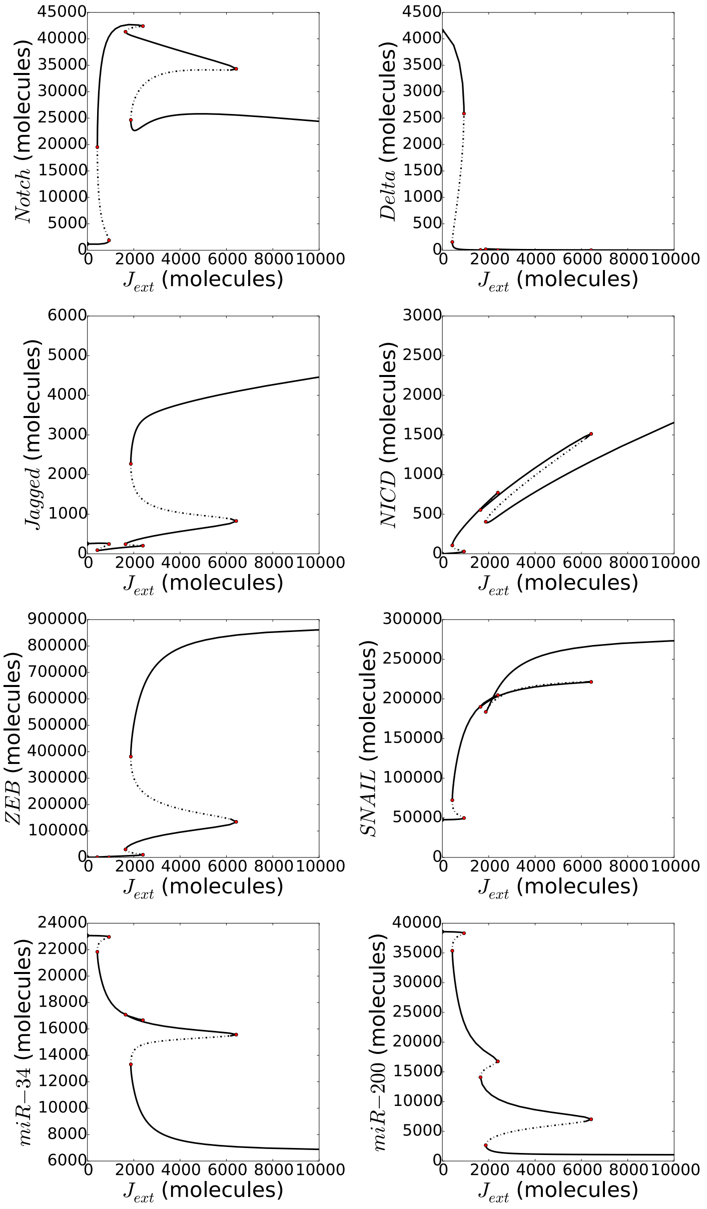

Figure S1 **Bifurcation curves of the levels of Notch (N), Delta (D), Jagged (J), NICD (I), Snail (S), Zeb (B), miR-34 (W) and miR-200 (Y) as a function of external Jagged in absence of Numb.** This plot shows the bifurcation diagrams of all the considered proteins and micro-RNAs corresponding to Fig. 1c.

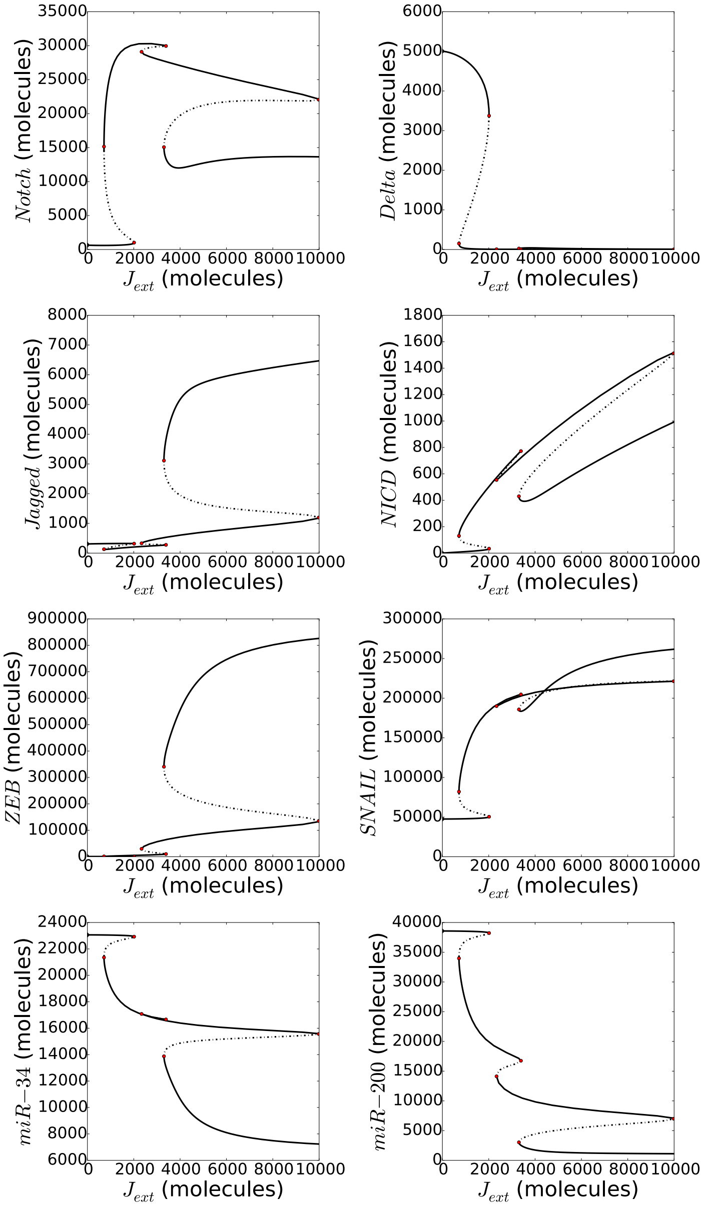

Figure S2 **Bifurcation curves of the levels of Notch (N), Delta (D), Jagged (J), NICD (I), Snail (S), Zeb (B), miR-34 (W) and miR-200 (Y) as a function of external Jagged in presence of Numb.** This plot shows the bifurcation diagrams of all the considered proteins and micro-RNAs corresponding to Fig. 1d.

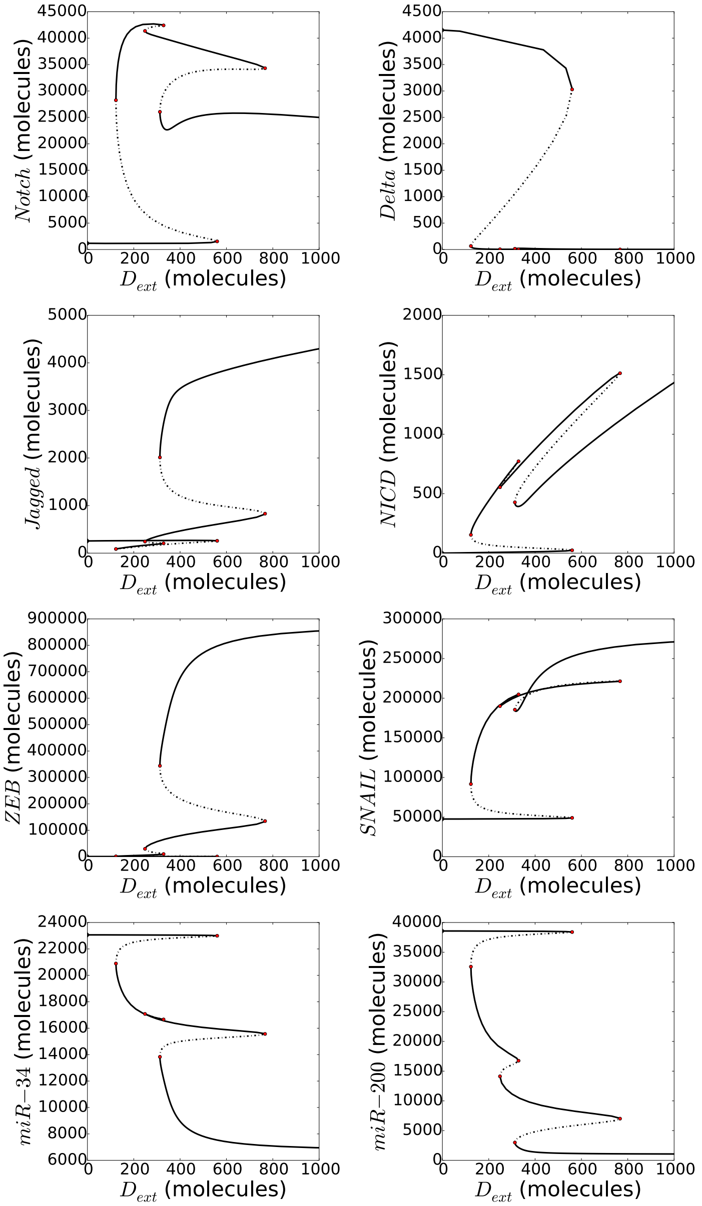

Figure S3 **Bifurcation curves of the levels of Notch (N), Delta (D), Jagged (J), NICD (I), Snail (S), Zeb (B), miR-34 (W) and miR-200 (Y) as a function of external Delta in absence of Numb.** This plot shows the bifurcation diagrams of all the considered proteins and micro-RNAs corresponding to Fig. 1e.

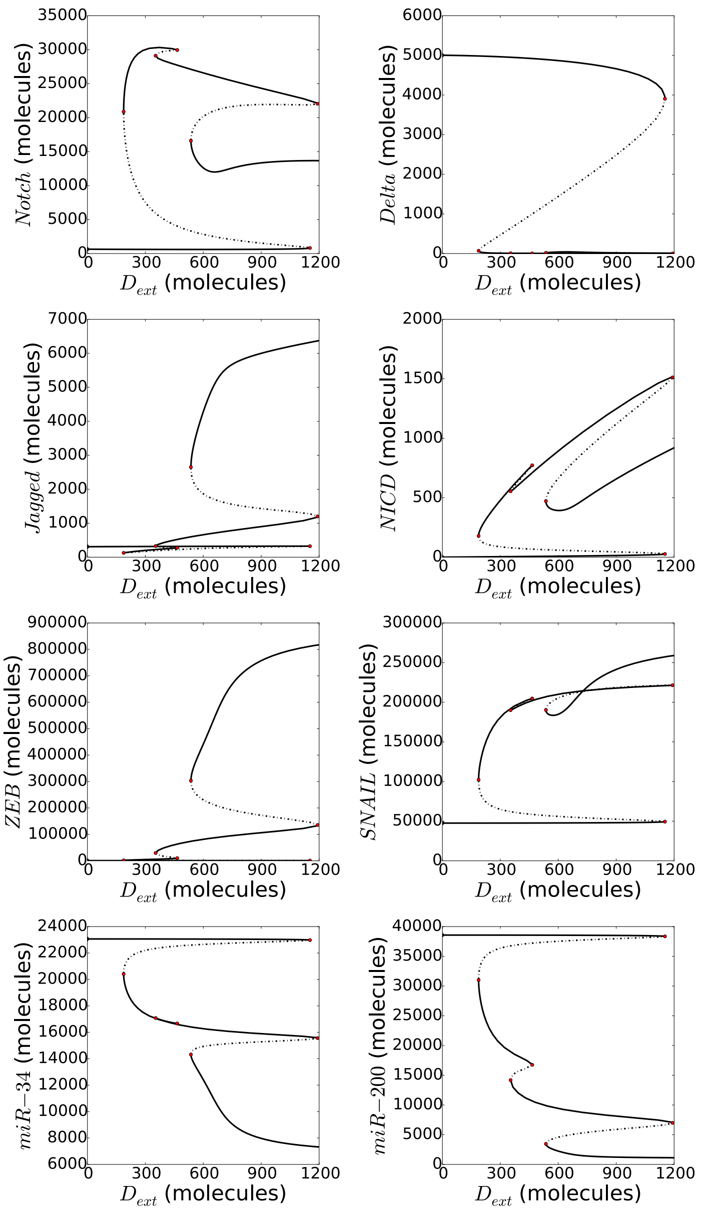

Figure S4 **Bifurcation curves of the levels of Notch (N), Delta (D), Jagged (J), NICD (I), Snail (S), Zeb (B), miR-34 (W) and miR-200 (Y) as a function of external Delta in presence of Numb.** This plot shows the bifurcation diagrams of all the considered proteins and micro-RNAs corresponding to Fig. 1f.

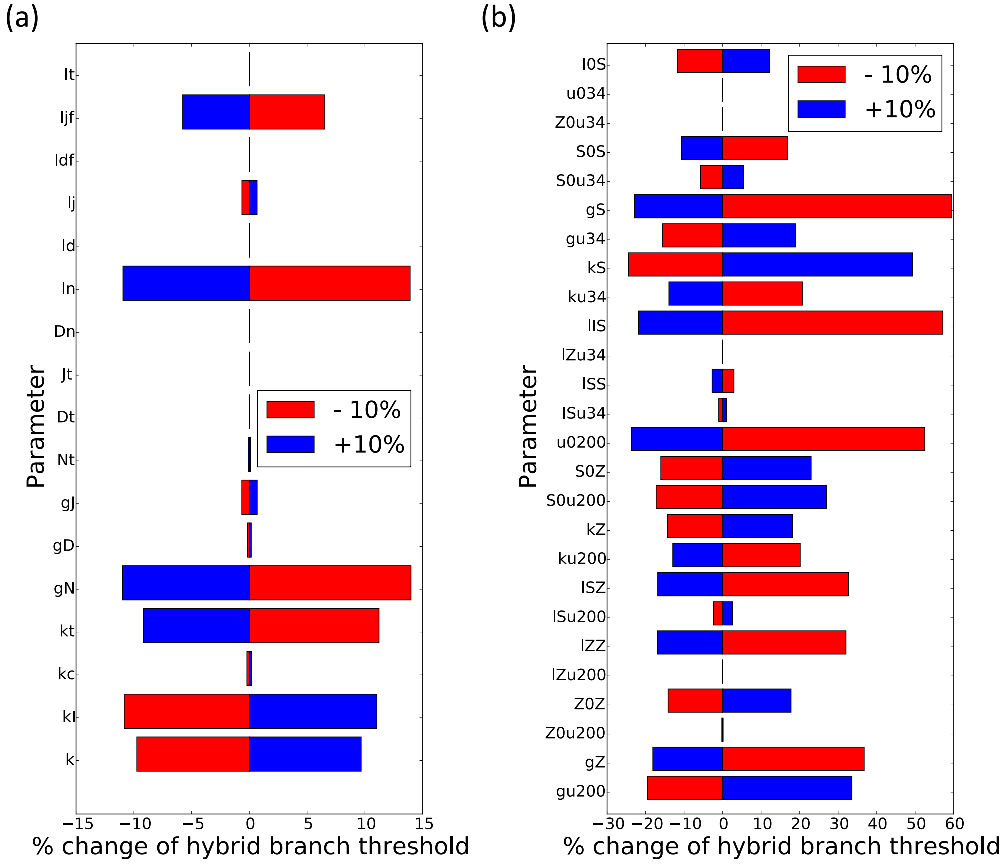

Figure S5 **Sensitivity of the single cell system to a 10% variation of the model's parameters.** (a) Percentage variation of the minimal value of Jext that allows for a stable hybrid E/M phenotype in Fig. 1d (i.e. the x-coordinate of the starting point of the hybrid E/M branch) upon 10% variation of the parameters of the original Notch-Delta-Jagged-NICD circuit. (b) Same as (a) for the parameters of the original EMT circuit.

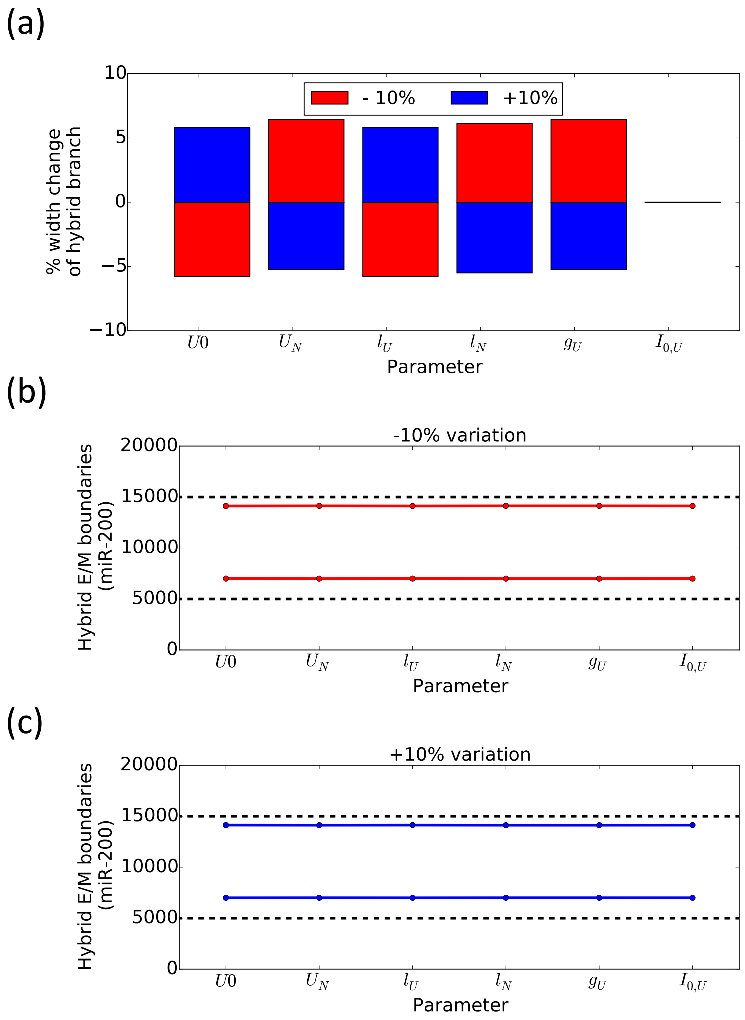

Figure S6 **Sensitivity of the model to a variation of the parameters related to the interactions of Numb.** (a) Percentage variation in the width of the interval of stability of the hybrid E/M phenotype upon 10% variation of the newly introduced parameters related to Numb. The interval of stability is defined as the interval that allows a stable E/M state in Fig. 1d. (b)-(c) Corresponding levels of the extremal values of miR-200 assumed by the E/M phenotype (at the beginning and end points of the hybrid E/M branch of Fig. 1d, respectively).

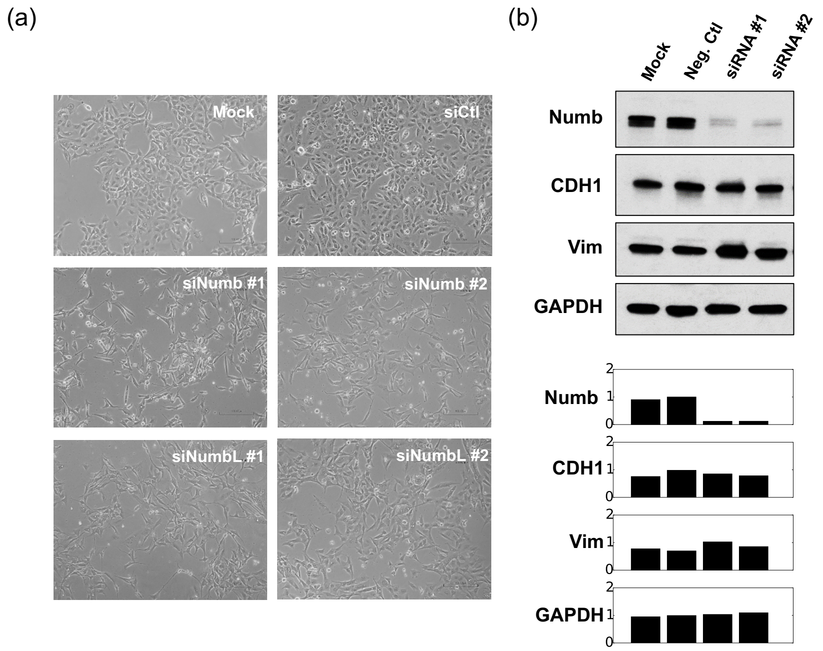

Figure S7 **Effect of Numb Knockdown in H1975 cells.** (a) Bright-field microscopy for mock H1975 cells, H1975 with control siRNA, and H1975 with siRNA against Numb or Numbl (100x magnification). (b) Western blot showing CDH1, VIM, Numb for the case of Numb knockdown.

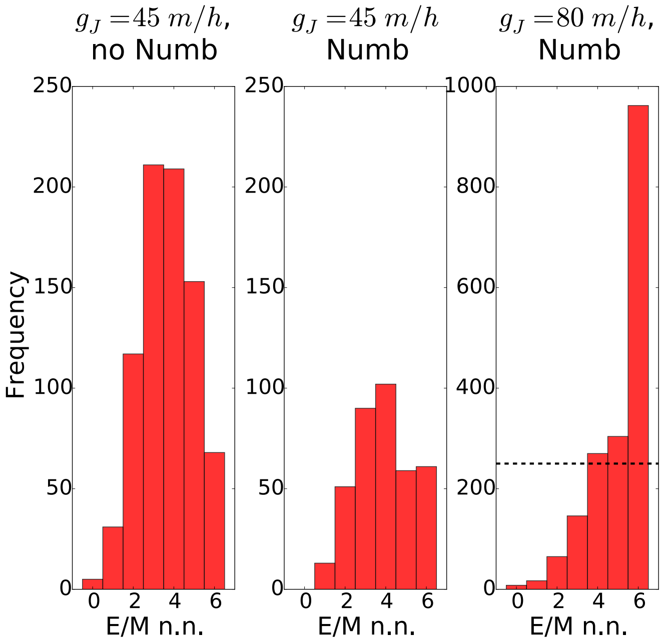

Figure S8 **Numb increases the number of hybrid cells that are surrounded by other hybrid cells.** Distribution of hybrid E/M nearest neighbours (E/M n. n.) of the hybrid E/M cells from the three snapshots of Fig. 3b-c-f. The dotted horizontal line for the case of g_J=80 m/h^{-1} (right) compared the scale of the y-axis with the first two histograms.

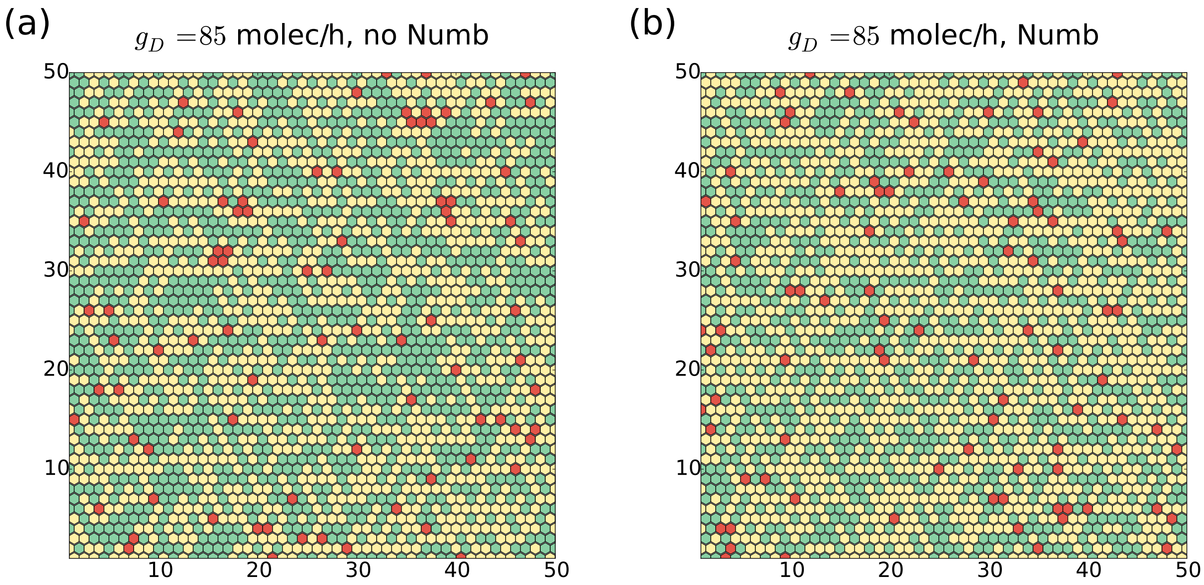

Figure S9 (a) Snapshot of the 2-dimensional cell layer for g_D=85, g_J=20 molecules h^{-1} in the absence of Numb. (b) Same as (a) in presence of Numb. Also, same as Fig. 4b, reported here for comparison with (a).

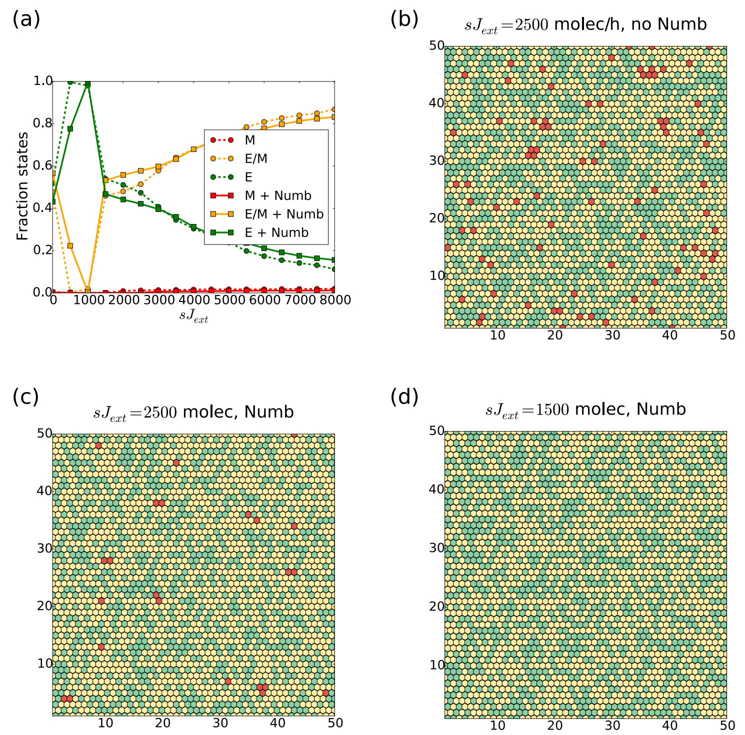

Figure S10 **Effect of Numb inhibition on Notch in presence of soluble Jagged when Notch signalling is Delta-dominated.** (a) Fraction of cells with different phenotypes (epithelial (E), epithelial/mesenchymal (E/M), mesenchymal (M)) as a function of the external concentration of soluble Jagged sJ_{ext} in a 2-dimensional 50x50 layer of cells in the absence or presence of Numb interactions (dashed and continuous lines, respectively) and the Notch signalling is Delta-dominated (g_J=20 molecules h^{-1}, g_D=85 molecules h^{-1}). (b) Snapshot of the 2-dimensional cell layer for sJ_{ext}=2500 molecules in absence of Numb. (c) Same as (b) in presence of Numb: the fraction of hybrid E/M cells is increased while mesenchymal cells are rare. (d) Snapshot for sJ_{ext}=1500 molecules) in presence of Numb: the hybrid-to-epithelial ratio of (b) is recovered at lower level of soluble Jagged, but there are no mesenchymal cells. Fractions of states and snapshots were measured after a transient of 120 h starting from the configuration of Fig. S8a and S8b as initial conditions for the case without and with Numb, respectively.

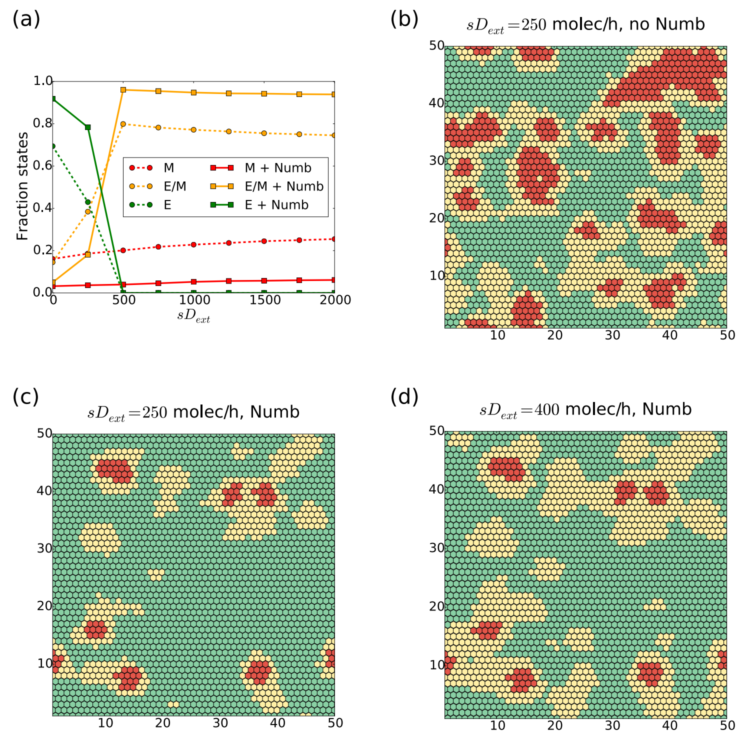

Figure S11 **Effect of Numb inhibition on Notch in presence of soluble Delta when Notch signalling is Jagged-dominated.** (a) Fraction of cells with different phenotypes (epithelial (E), epithelial/mesenchymal (E/M), mesenchymal (M)) as a function of the external concentration of soluble Delta sD_{ext} in a 2-dimensional 50x50 layer of cells in the absence or presence of Numb interactions (dashed and continuous lines, respectively and the Notch signalling is Jagged-dominated (g_J=45 molecules h^{-1}, g_D=20 molecules h^{-1})). (b) Snapshot of the 2-dimensional cell layer for sD_{ext}=250 molecules in absence Numb. (c) Same as (b) in presence of Numb: the fraction of hybrid E/M cells is decreased and mesenchymal cells are rare. Overall, the E/M cluster formation is considerably reduced. (d) Snapshot for sJ_{ext}=400 molecules in presence of Numb: the high occurrence of E/M cells typical of (b) is recovered at a higher level of soluble Jagged, but the fraction of mesenchymal cells is considerably decreased. Fractions of states and snapshots were measured after a transient of 120 h starting from the configuration of Fig. 4b and 4c as initial conditions for the case without and with Numb, respectively.

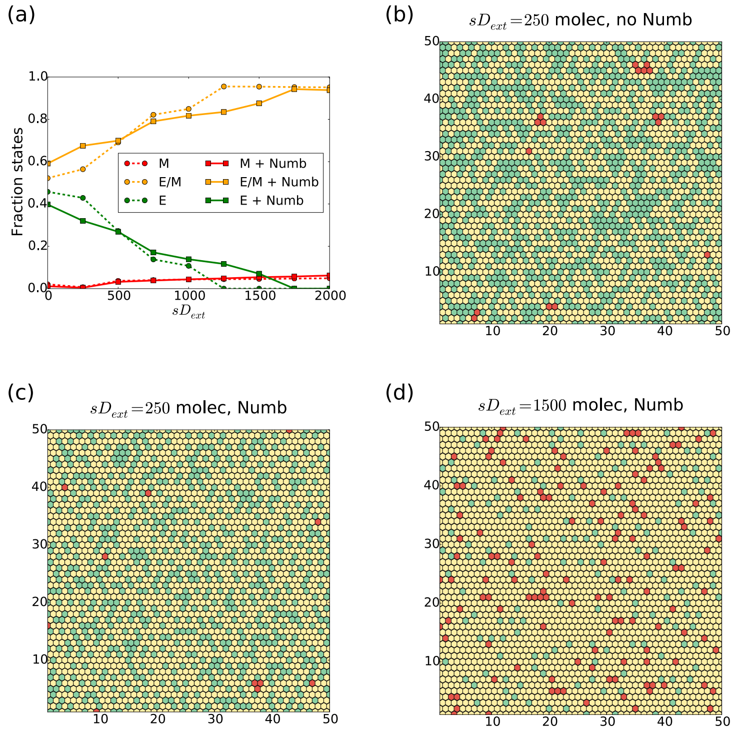

Figure S12 **Effect of Numb inhibition on Notch in presence of soluble Delta when Notch signalling is Delta-dominated.** (a) Fraction of cells with different phenotypes (epithelial (E), epithelial/mesenchymal (E/M), mesenchymal (M)) as a function of the external concentration of soluble Delta sD_{ext} in a 2-dimensional 50x50 layer of cells in the absence or presence of Numb interactions (dashed and continuous lines, respectively) and the Notch signalling is Delta-dominated (g_J=20 molecules h^{-1}, g_D=85 molecules h^{-1}). (b) Snapshot of the 2-dimensional cell layer for sD_{ext}=250 molecules in absence of Numb. (c) Same as (b) with Numb: the fraction of hybrid E/M cells is increased but their "salt and pepper" distribution is conserved. (d) Snapshot for sJ_{ext}=1500 molecules in presence of Numb: at high concentrations of soluble Delta most cells undergo partial EMT. Fractions of states and snapshots were measured after a transient of 120 h starting from the configuration of Fig. S8a and S8b as initial conditions for the case without and with Numb, respectively.

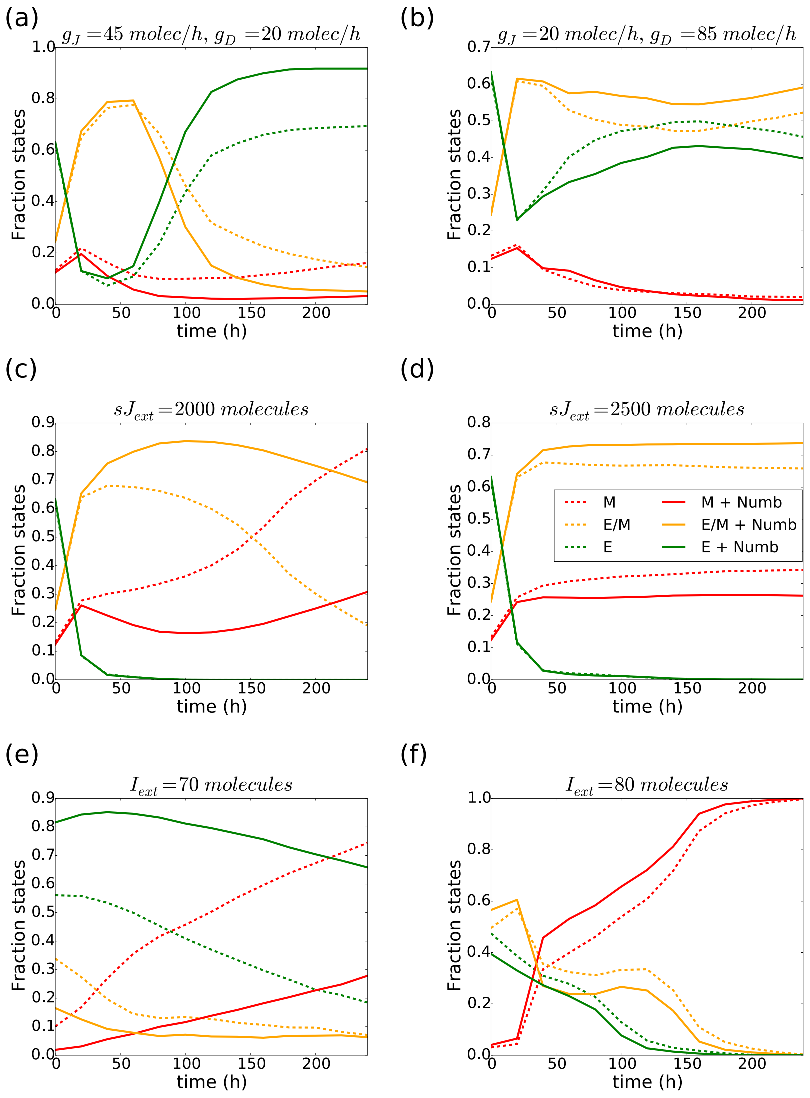

Figure S13 **Effect of Numb on the time dynamics of the fractions of states under various conditions.** Time trajectories of the fraction of states in absence of Numb (dashed lines) and in presence of Numb (continuous lines) in a 2-dimensional layer of 50x50 cells. Left plots (a-c-e) depict dynamics with Jagged-dominated Notch signalling (g_J=45 molecules h^{-1}, g_D=20 molecules h^{-1}), right plots (b-d-f) depict the case of Delta-dominated Notch-signalling g_J=20 molecules h^{-1}, g_D=85 molecules h^{-1}). (a)-(b) No external induction. (c)-(d) Cells are exposed to soluble Jagged. (e)-(f) Cells are exposed to an external EMT inducer (I_{ext}=70 molecules for e, I_{ext}=80 molecules for f). Trajectories in (a), (b), (c) and (d) start from the same initial conditions. Each trajectory in (e) and (f) starts from its corresponding configuration after 120 hours with the corresponding levels of Delta/Jagged and without/with Numb, but without inducer (Fig. S14b and S14c for (e) and Fig. S15b and S15c for (f)).

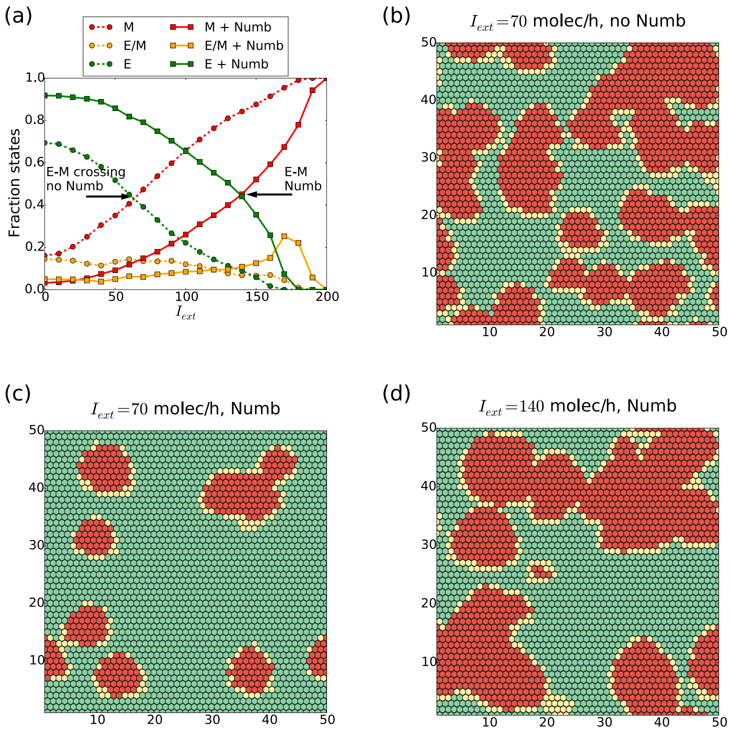

Figure S14 **Effect of Numb inhibition on Notch in presence of external EMT inducer when Notch signalling is Jagged-dominated.** (a) Fraction of cells with different phenotypes (epithelial (E), epithelial/mesenchymal (E/M), mesenchymal (M)) as a function of the external concentration of EMT inducer I_{ext} in a 2-dimensional 50x50 layer of cells in absence or presence of Numb interactions (dashed and continuous lines, respectively) and the Notch signalling is Jagged-dominated (g_J=45 molecules h^{-1}, g_D=20 molecules h^{-1}). At any given value of I_{ext} Numb decreases the fraction of mesenchymal cells and increases the fraction of epithelial cells. (b) Snapshot of the 2-dimensional cell layer for I_{ext} =70 molecules in absence of Numb. (c) Same as (b) with Numb: the fraction of mesenchymal cells is drastically decreased. (d) Snapshot for I_{ext} =140 molecules in presence of Numb: a spatial pattern similar to that of (b) is recovered. Fractions of states and snapshots were measured after a transient of 120 h starting from the configuration of Fig. 5b and 5c as initial conditions for the case without and with Numb, respectively.

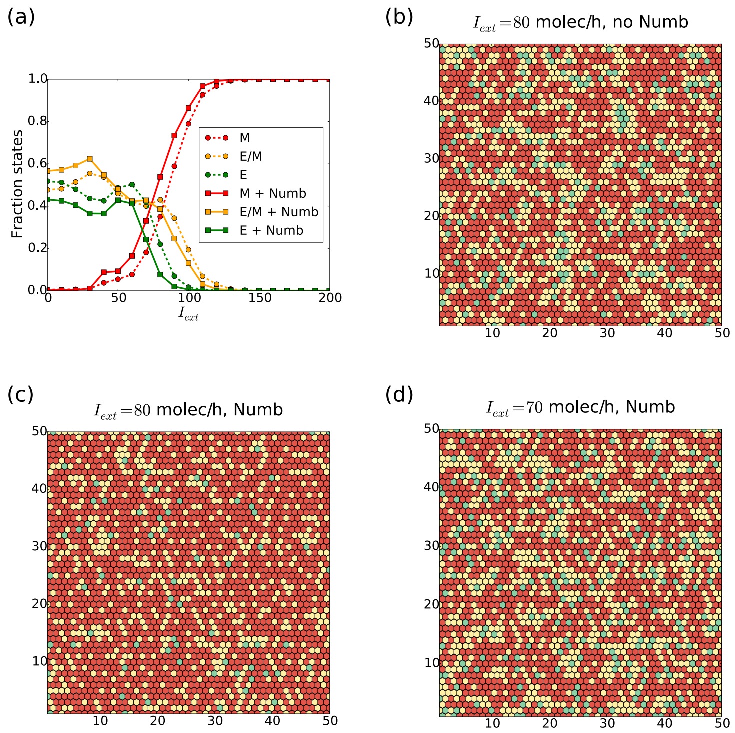

Figure S15 **Effect of Numb inhibition on Notch in presence of external EMT inducer when Notch signalling is Delta-dominated.** (a) Fraction of cells with different phenotypes (epithelial (E), epithelial/mesenchymal (E/M), mesenchymal (M)) as a function of the external concentration of EMT inducer I_{ext} in a 2-dimensional 50x50 layer of cells in absence or presence of Numb (dashed and continuous lines, respectively) and the Notch signalling is Delta-dominated (g_J=20 molecules h^{-1}, g_D=85 molecules h^{-1}). For any given value of I_{ext}, Numb slightly increases the fraction of mesenchymal cells and decreases the fraction of epithelial cells. (b) Snapshot of the 2-dimensional cell layer for I_{ext} =80 molecules in absence of Numb. (c) Same as (b) in presence of Numb: the fraction of mesenchymal cells is increased while the fraction of epithelial cells is decreased. The abundance of the hybrid E/M cells is similar. (d) Snapshot for I_{ext} =70 molecules in presence of Numb: a spatial pattern similar to that of (b) is recovered. Fractions of states and snapshots were measured after a transient of 120 h starting from the configuration of Fig. S8a and S8b as initial conditions for the case without and with Numb, respectively.

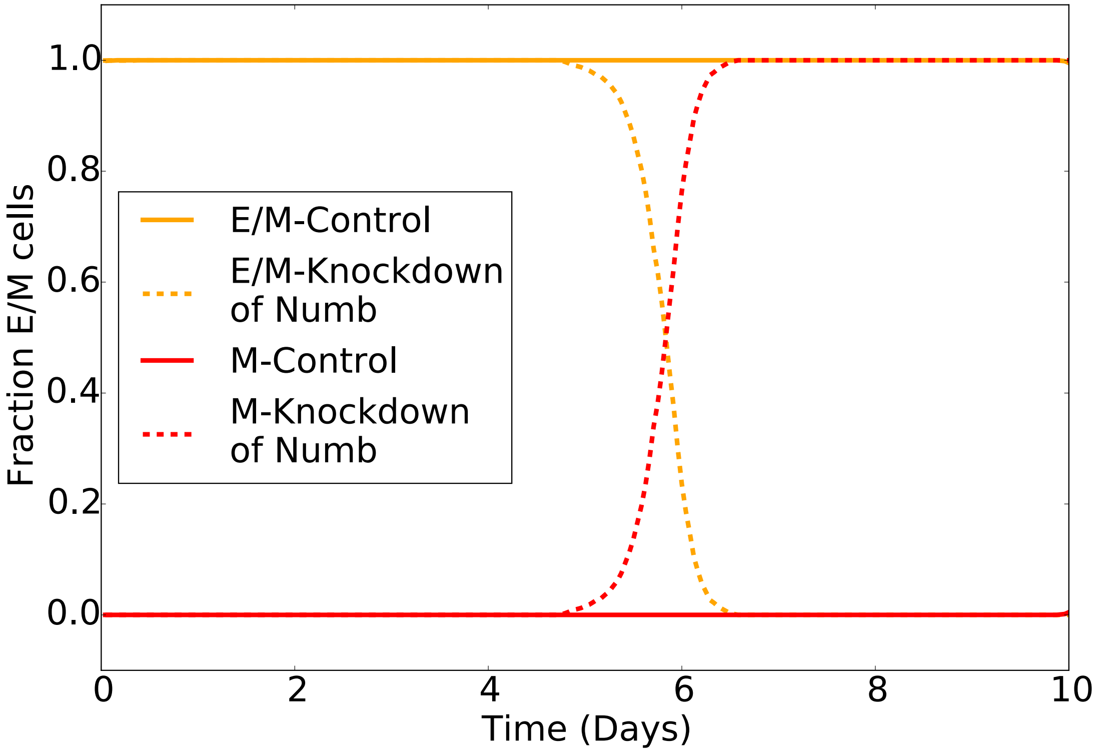

Figure S16 Temporal dynamics of the fraction of hybrid E/M (yellow) and mesenchymal M (red) cells in a two-dimensional layer of 50x50 cells for a strong Notch-Jagged signalling (g_J=80 molecules h^{-1}, g_D=20 molecules h^{-1}) and in presence of a medium level of EMT-inducer (I_{ext} =30 molecules). Initial condition of the level of all micro-RNAs, TFs and proteins are random but within the range allowed in the hybrid E/M phenotype (see Fig. S2-S5). In the case of Numb-Notch-EMT circuit all cells remain hybrid, while for the Numb-knockdown (dotted curves) all cells become mesenchymal within 5-6 days.

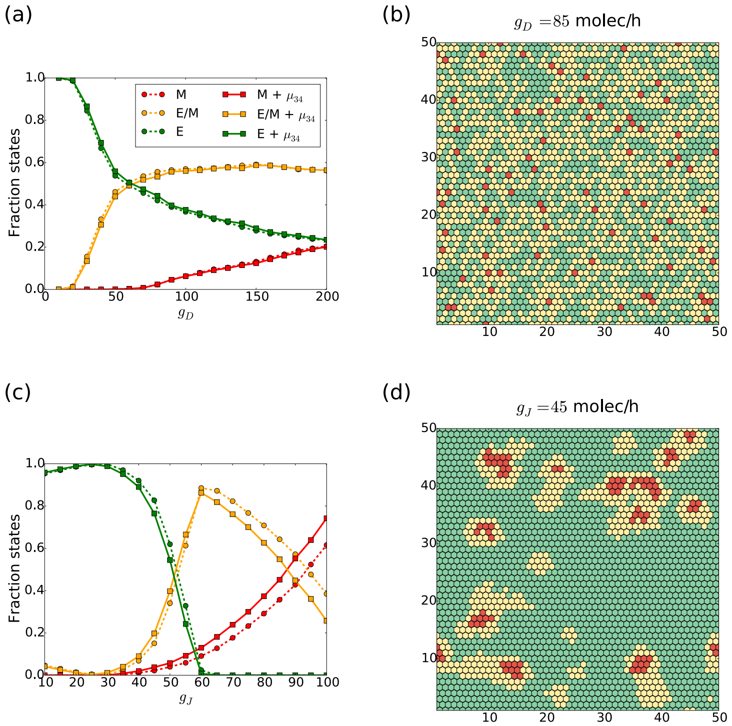

Figure S17 **Effect of the inhibition of Numb by miR-34.** (a) Fraction of cells with different phenotypes (epithelial (E), epithelial/mesenchymal (E/M), mesenchymal (M)) as a function of the production rate of the ligand Delta g_D in a 2-dimensional 50x50 layer of cells when the inhibition of miR-34 on Numb is included in the model. Dotted curves stand for the case where Numb is in the circuit but miR-34 does not inhibit it, continuous curves for the case when miR-34 inhibits Numb. (b) Snapshot of the 2-dimensional cell layer for g_D=85 molecules h^{-1}. (c) Same as (a) as a function of Jagged production rate g_J. (d) Snapshot of the 2-dimensional cell layer for g_J=45 molecules h^{-1}. Fractions of states and snapshots were measured after a transient of 120 h starting from the same initial conditions. For (a) and (b), the production rate of Jagged is g_J=20 molecules h^{-1}. For (c) and (d), the rate of production of Delta is g_D=20 molecules h^{-1}.

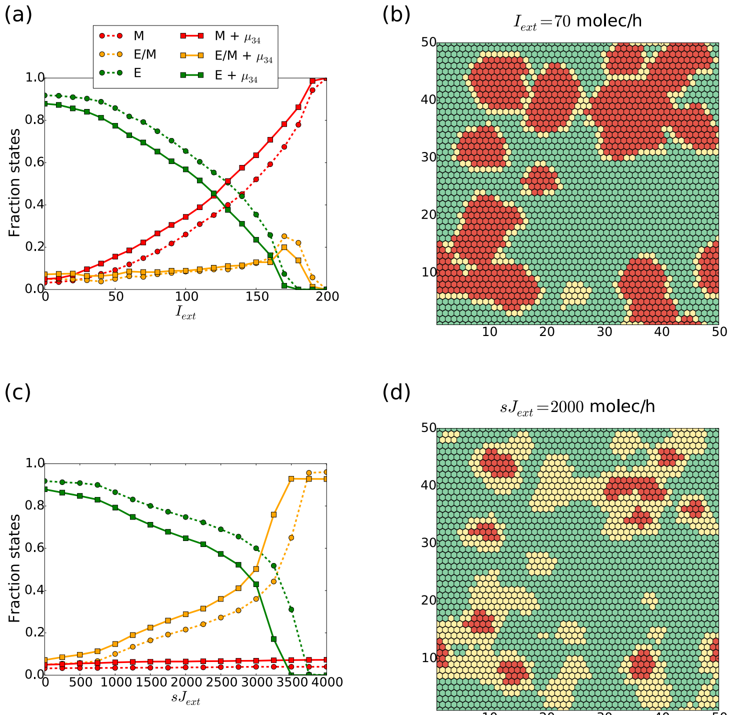

Figure S18 **Effect of miR-34 inhibition on Numb in presence of external EMT inducer and soluble Jagged when Notch signalling is Jagged-dominated.** (a) Fraction of cells with different phenotypes (epithelial (E), epithelial/mesenchymal (E/M), mesenchymal (M)) as a function of the external concentration of EMT inducer I_{ext} in a 2-dimensional 50x50 layer of cells when the inhibition of miR-34 on Numb is included in the model and the Notch signalling is Jagged-dominated (g_J=45 molecules h^{-1}, g_D=20 molecules h^{-1}). Dotted curves stand for the case where Numb is in the circuit but miR-34 does not inhibit it, continuous curves for the case when miR-34 inhibits Numb. (b) Snapshot of the 2-dimensional cell layer for I_{ext}=70 molecules. (c) Same as (a) as a function of the external concentration of soluble Jagged sJ_{ext}. (d) Snapshot of the 2-dimensional cell layer for sJ_{ext}=2000 molecules. Fractions of states and snapshots were measured after a transient of 120 h starting from the same initial conditions.

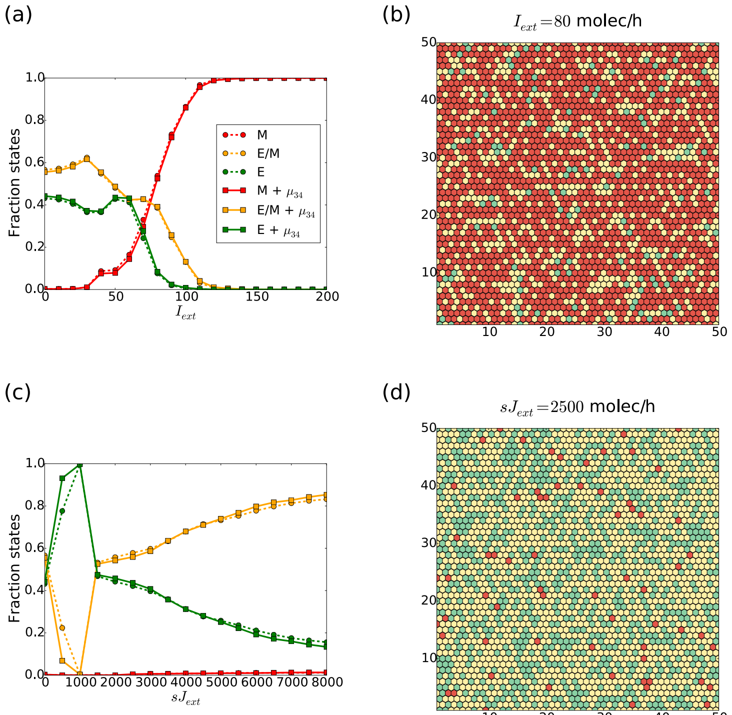

Figure S19 **Effect of miR-34 inhibition on Numb in presence of external EMT inducer and soluble Jagged when Notch signalling is Delta-dominated.** (a) Fraction of cells with different phenotypes (epithelial (E), epithelial/mesenchymal (E/M), mesenchymal (M)) as a function of the external concentration of EMT inducer I_{ext} in a 2-dimensional 50x50 layer of cells when the inhibition of miR-34 on Numb is included in the model and the Notch signalling is Delta-dominated (g_J=20 molecules h^{-1}, g_D=85 molecules h^{-1}). Dotted curves stand for the case where Numb is in the circuit but miR-34 does not inhibit it, continuous curves for the case when miR-34 inhibits Numb. (b) Snapshot of the 2-dimensional cell layer for I_{ext}=80 molecules. (c) Same quantity of (a) as a function of the external concentration of soluble Jagged sJ_{ext}. (d) Snapshot of the 2-dimensional cell layer for sJ_{ext}=2500 molecules. Fractions of states and snapshots were measured after a transient of 120 h starting from the same initial conditions.

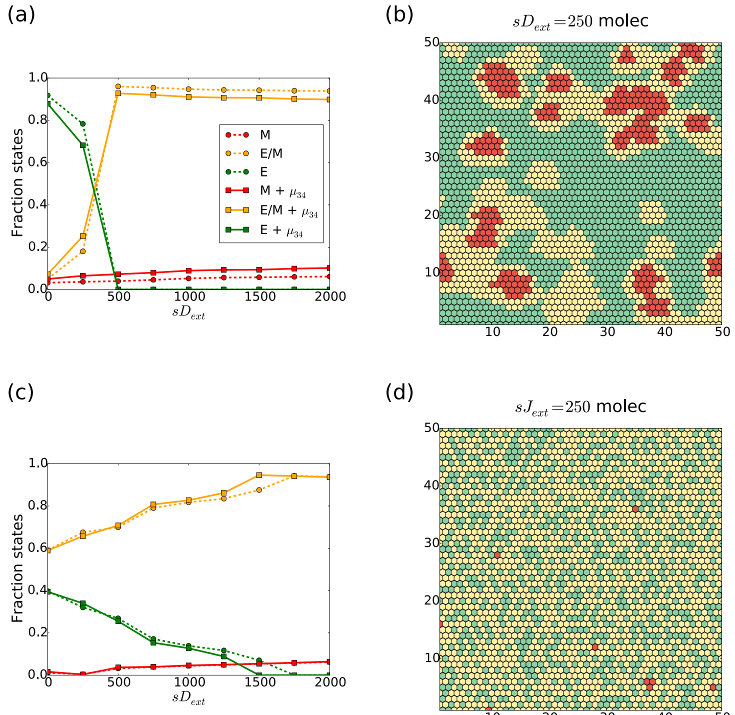

Figure S20 **Effect of miR-34 inhibition on Numb in presence of soluble Delta.** (a) Fraction of cells with different phenotypes (epithelial (E), epithelial/mesenchymal (E/M), mesenchymal (M)) as a function of the external concentration of soluble Delta sD_{ext} in a 2-dimensional 50x50 layer of cells when the inhibition of miR-34 on Numb is included in the model and the signalling is Jagged-dominated (g_J=45 molecules h^{-1}, g_D=20 molecules h^{-1}). Dotted curves stand for the case where Numb is in the circuit but miR-34 does not inhibit it, continuous curves for the case when miR-34 inhibits Numb. (b) Snapshot of the 2-dimensional cell layer for sD_{ext}=250 molecules from (a). (c) Same as (a) when the signalling is Delta-dominated (g_J=20 molecules h^{-1}, g_D=85 molecules h^{-1}). (d) Snapshot of the 2-dimensional cell layer for sD_{ext}=250 molecules from (c). Fractions of states and snapshots were measured after a transient of 120 h starting from the same initial conditions.

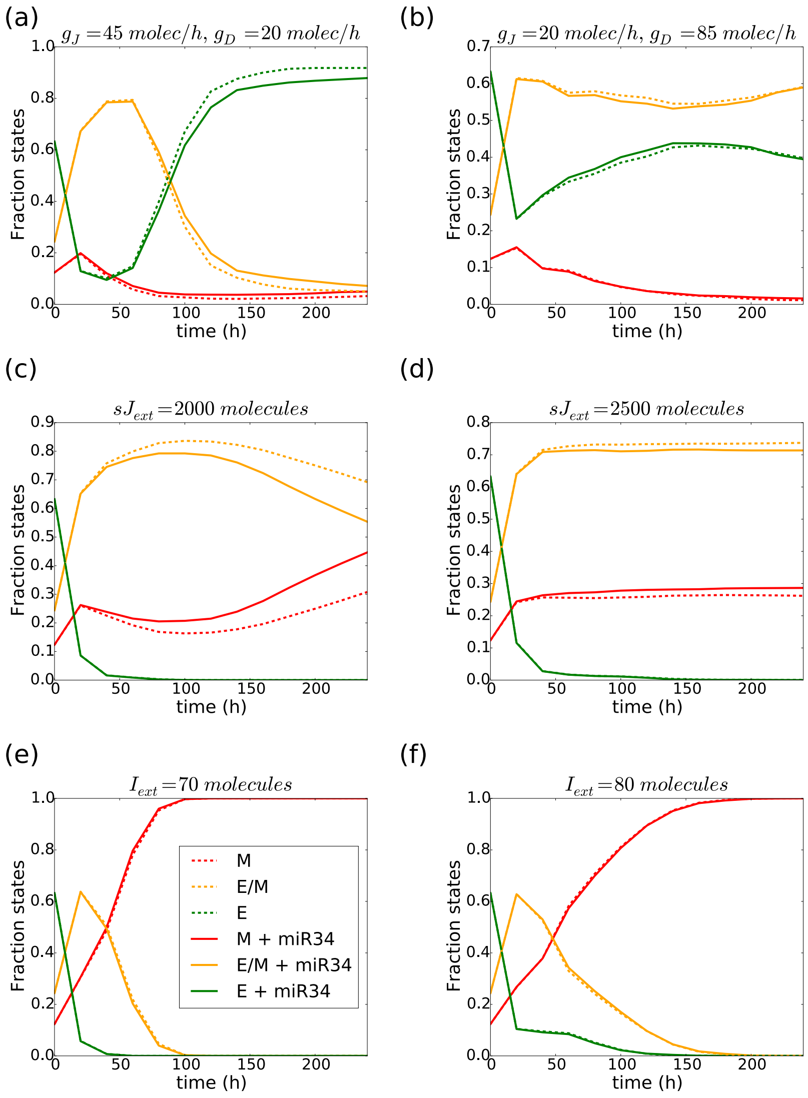

Figure S21 **Effect of miR-34 inhibition on Numb on the time dynamics of the fractions of states under various conditions.** Time trajectories of the fraction of states for the case where Numb is in the circuit but miR-34 does not inhibit it (dashed lines) and for the case when miR-34 inhibits Numb (continuous lines) in a 2-dimensional layer of 50x50 cells Left plots (a-c-e) depict dynamics with Jagged-dominated Notch signalling (g_J=45 molecules h^{-1}, g_D=20 molecules h^{-1}), right plots (b-d-f) show the case of Delta-dominated Notch-signalling g_J=20 molecules h^{-1}, g_D=85 molecules h^{-1}). (a)-(b) No external signal. (c)-(d) Cells are exposed to soluble Jagged. (e)-(f) Cells are exposed to an external EMT inducer. All trajectories start from the same initial conditions.

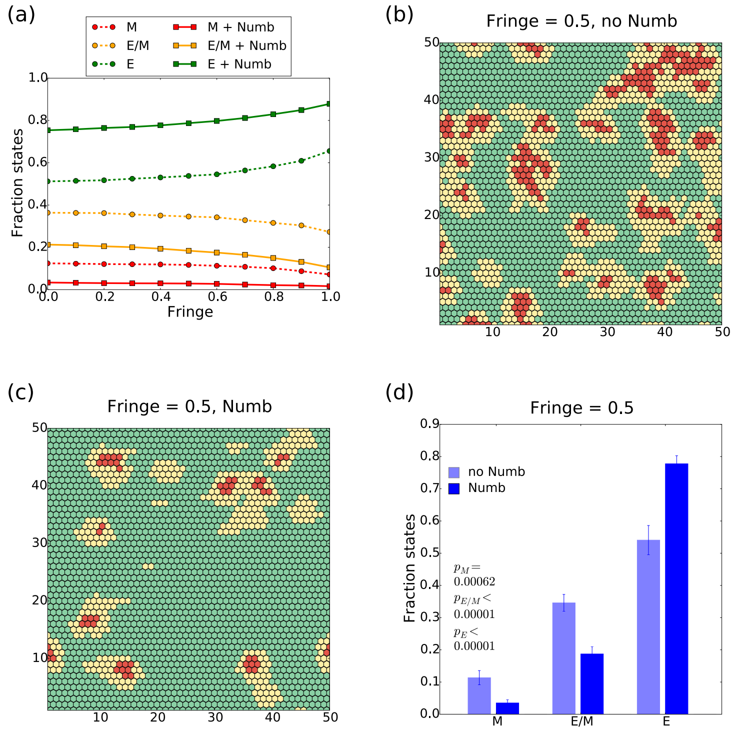

Figure S22 **Effect of Numb and Fringe when Notch signalling is Jagged-dominated.** (a) Fraction of cells with different phenotypes (epithelial (E), epithelial/mesenchymal (E/M), mesenchymal (M)) as a function of the Fringe regulation on notch-delta and notch-jagged interactions in a 2-dimensional 50x50 layer of cells in absence or presence of Numb (dashed and continuous lines, respectively) and the Notch signalling is Jagged-dominated (g_J=45 molecules h^{-1}, g_D=20 molecules h^{-1}). (b) Snapshot of the 2-dimensional cell layer for an intermediate Fringe effect fng=0.5 in absence of Numb. (c) Same as (b) with Numb: the fraction of hybrid E/M cells is decreased and the occurrence of clusters is considerably limited by Numb. (d) Average fraction of cells in the three states for fng=0.5: Numb diminishes both partial and complete EMT. Averages are computed over 10 simulations starting from different randomly chosen phenotype distributions and the fractions of states and snapshots were measured after a transient of 120 h.

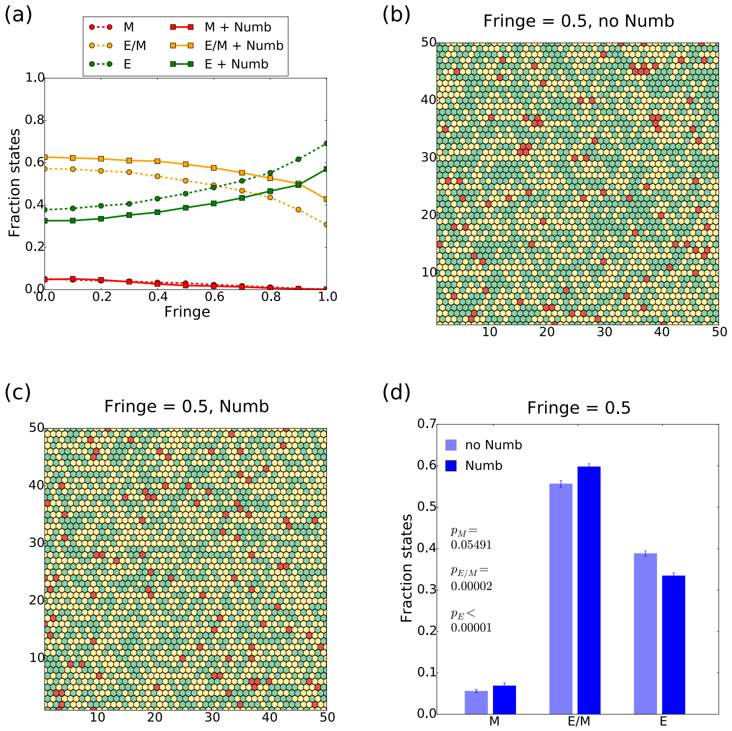

Figure S23 **Effect of Numb and Fringe when Notch signalling is Delta-dominated.** (a) Fraction of cells with different phenotypes (epithelial (E), epithelial/mesenchymal (E/M), mesenchymal (M)) as a function of the Fringe regulation on notch-delta and notch-jagged interactions in a 2-dimensional 50x50 layer of cells in absence or presence of Numb (dashed and continuous lines, respectively) and the Notch signalling is Delta-dominated (g_J=20 molecules h^{-1}, g_D=85 molecules h^{-1}). (b) Snapshot of the 2-dimensional cell layer for an intermediate Fringe effect fng=0.5 in absence of Numb. (c) Same as (b) with Numb: the fraction of hybrid E/M cells is slightly increased and the "salt-and-pepper" pattern is conserved. (d) Average fraction of cells in the three states for fng=0.5: Numb slightly favourites the hybrid phenotype over the epithelial phenotype. Averages are computed over 10 simulations starting from different randomly chosen phenotype distributions and the fractions of states and snapshots were measured after a transient of 120 h.

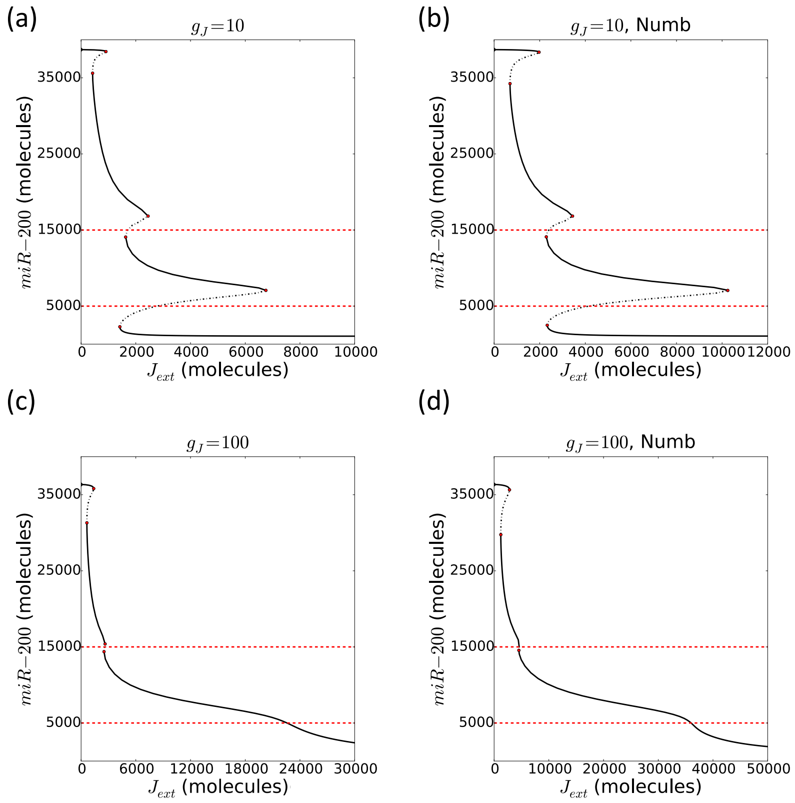

Figure S24 **Thresholds for the E, E/M and M phenotypes for extremal parameter values**. Bifurcation diagram of miR-200 vs the external level of Jagged ligand Jext for (a) gJ=10, (b) gJ=10+Numb, (c) gJ=100, (d) gJ=100+Numb. Red dotted lines highlight the threshold used to distinguish the phenotypes (miR200 > 15000: E, 15000 > miR200 > 5000: E/M, miR200 < 5000: M).

Movie Captions

M1: Temporal dynamics of the cell phenotype distribution (green: epithelial, yellow: hybrid E/M, red: mesenchymal) over 120 hours for the 50x50 layer of cells in the Control case (no Numb) and with weak Notch-Jagged signalling. The final configuration of this simulation (120 hours) corresponds to the distribution presented in Fig. 3b. For this simulation, the parameters are the same of Fig. 3b.

M2: Temporal dynamics of the cell phenotype distribution (green: epithelial, yellow: hybrid E/M, red: mesenchymal) over 120 hours for the 50x50 layer of cells in the Numb-dependent case and with weak Notch-Jagged signalling. The final configuration of this simulation (120 hours) corresponds to the distribution presented in Fig. 3c. For this simulation, the parameters are the same of Fig. 3c.

M3: Temporal dynamics of the cell phenotype distribution (green: epithelial, yellow: hybrid E/M, red: mesenchymal) over 120 hours for the 50x50 layer of cells in the Control case (no Numb) and with strong Notch-Jagged signalling. The final configuration of this simulation (120 hours) corresponds to the distribution presented in Fig. 3e. For this simulation, the parameters are the same of Fig. 3e.

M4: Temporal dynamics of the cell phenotype distribution (green: epithelial, yellow: hybrid E/M, red: mesenchymal) over 120 hours for the 50x50 layer of cells in the Numb-dependent case and with strong Notch-Jagged signalling. The final configuration of this simulation (120 hours) corresponds to the distribution presented in Fig. 3f. For this simulation, the parameters are the same of Fig. 3f.

M5: Temporal dynamics of the cell phenotype distribution (green: epithelial, yellow: hybrid E/M, red: mesenchymal) over 120 hours for the 50x50 layer of cells in the Control case (no Numb) and with Notch-Delta signalling. The final configuration of this simulation (120 hours) corresponds to the distribution presented in Fig. S9a. For this simulation, the parameters are the same of Fig. S9a.

M6: Temporal dynamics of the cell phenotype distribution (green: epithelial, yellow: hybrid E/M, red: mesenchymal) over 120 hours for the 50x50 layer of cells in the Numb-dependent case and with Notch-Delta signalling. The final configuration of this simulation (120 hours) corresponds to the distribution presented in Fig. 4b (or S9b). For this simulation, the parameters are the same of Fig. 4b.
